# Analysis of control and computational strategies for green energy integration for sociotechnical ecological power infrastructure in Indian and African markets

**DOI:** 10.1038/s41598-025-96773-2

**Published:** 2025-04-22

**Authors:** Prince Kumar, Kunal Kumar, Nabanita Adhikary, Eshet Lakew Tesfaye

**Affiliations:** 1 Department of Electrical Engineering, National Institute of Technology Silchar, Silchar, Assam India; 2 Department of Electrical Engineering, National Institute of Technology Rourkela, Rourkela, Odisha India; 3 Department of Biotechnology, Hawassa University, Hawassa, Ethiopia

**Keywords:** Control techniques, Green energy, Optimization, Computational methodologies, Wind, Nuclear, Energy science and technology, Mathematics and computing

## Abstract

The rapid expansion of energy infrastructure in emerging economies, particularly in India and Africa, necessitates advanced control and computational strategies to ensure the seamless integration of green energy resources with conventional power systems. This study conducts a comprehensive analysis of state-of-the-art control mechanisms and optimization techniques for hybrid power networks, focusing on enhancing grid stability, frequency regulation, and resilience under dynamic loading and climatic variations. It explores advanced generation control strategies, including adaptive and predictive control frameworks, to mitigate the inherent intermittency of renewable energy sources. Furthermore, the paper examines multi-objective optimization methodologies for energy dispatch, frequency stabilization, and reliability enhancement in multi-entity power networks. By proposing a robust and computationally efficient framework for hybrid energy integration, this study contributes to the development of resilient, self-sustaining power systems crucial for ensuring long-term energy security, operational efficiency, and economic growth in rapidly developing regions.

## Introduction

The modern electrical grid has undergone a profound transformation, evolving into a sophisticated and expansive network. This transformation has been driven primarily by the continually increasing scale of the grid and the sporadic fluctuations in the demand for electricity. The dynamic nature of these factors has given rise to a series of challenges, with a paramount focus on ensuring the stability of power network frequency and the efficient flow of power between various control regions under standard operating conditions^1,2^.

**Fig. 1:**
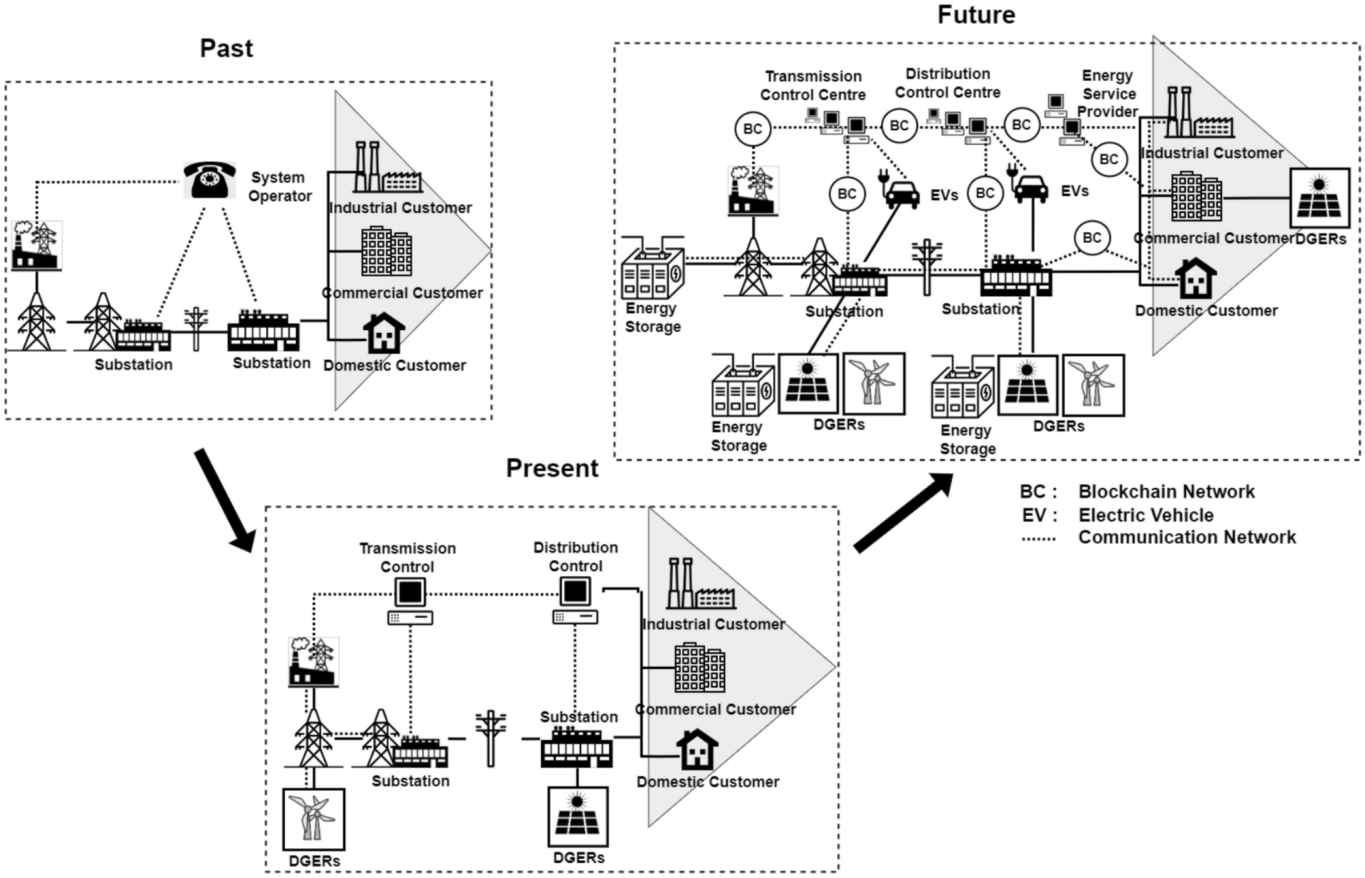
Evolution of the electrical grid from centralized to decentralized with blockchain integration and advanced control systems.

Figure 1 illustrates this transformation, highlighting the grid’s evolution from a simple and linear system in the past to a more sophisticated and interconnected network in the present and future. In the past, the grid was largely centralized, with power flowing unidirectionally from generation sources through substations to industrial, commercial, and domestic consumers. Control was managed manually with minimal technological integration. Moving to the present, the grid has become more dynamic, integrating Distributed Generation Energy Resources (DGERs) like solar, wind as well as storage systems that help balance supply and demand. Transmission and distribution are now controlled digitally, allowing for better real-time management. Looking into the future, the grid is expected to incorporate advanced technologies such as blockchain networks (BC) for secure energy transactions, and electric vehicles (EVs) as both consumers and suppliers of energy. This future grid will be highly decentralized, with energy flow managed through sophisticated communication networks and control systems, ensuring resilience and responsiveness to demand fluctuations.

One of the central challenges arises from the inherent unpredictability and variability in electricity demand on the consumer side^3^. The grid must contend with abrupt fluctuations in demand, leading to an intrinsic imbalance between the load requirements of consumers and the actual power generated by the system. This imbalance, in turn, triggers variations in system frequency, which is a critical parameter for the smooth functioning of the electrical grid. As a consequence of these frequency variations, there is a dynamic exchange of power across tie-lines that connect different control areas within the grid. Control areas are distinct regions where the balance between power generation and consumption is actively managed. The exchange of power between these control areas is essential for maintaining a balance in the overall grid operation.

The challenges posed by the imbalance between load and power generation underscore the need for advanced control mechanisms and technologies. These mechanisms are crucial for optimizing power distribution, minimizing disruptions, and ensuring the overall stability and reliability of the electrical grid. The Fig. 1 reinforces the importance of these advanced control systems, showing how the grid’s evolution has included increasingly complex and integrated control centres. In the future grid, these control systems will be essential for managing the diverse and decentralized energy resources, ensuring stable and efficient power flow across the entire network.

To further enhance the resilience and responsiveness of the modern electrical grid, integrating a diverse mix of energy sources is essential. Among these, green energy sources such as solar, wind, hydropower, geothermal, and biomass play a critical role in ensuring sustainability and reducing environmental impact. In addition, nuclear energy, with its capacity for providing consistent, large-scale power with minimal greenhouse gas emissions, presents a valuable, albeit complex, component of the energy portfolio. By diversifying energy sources and incorporating advanced technologies, the grid can better manage the inherent challenges of demand variability and power system stability, paving the way for a greener and more reliable energy future^4,5^. A study on environmental impacts with renewable energy sources is presented in^6^ by authors.

In summary, the evolution of the electrical grid into a complex network is a response to the increasing scale and demand variability. The resulting challenges, particularly in maintaining power system stability and efficient power flow, necessitate ongoing advancements in control systems and technologies to ensure a resilient and responsive electrical grid in today’s dynamic energy landscape.

### Major contributions

This study presents a comprehensive analysis of advanced control and computational strategies for integrating green energy sources into hybrid power systems, with a particular focus on Indian and African markets. The major contributions of this research are as follows:

1. Advanced Control Strategies for Green Energy Integration: The study develops and evaluates state-of-the-art generation control mechanisms, including adaptive and predictive control frameworks, to mitigate the inherent intermittency of renewable energy sources such as solar and wind. These strategies improve grid stability, frequency regulation, and resilience under dynamic loading and climatic variations.
2. Optimization Techniques for Hybrid Power Networks: A multi-objective optimization framework is proposed for energy dispatch, frequency stabilization, and reliability enhancement in multi-entity hybrid power networks. This includes the application of metaheuristic algorithms such as Firefly Optimization (FFO), Particle Swarm Optimization (PSO), and Teaching–Learning-Based Optimization (TLBO) to fine-tune control parameters and ensure the efficient operation of power systems.
3. Hybrid Energy System Modeling and Economic Viability: The research critically examines the integration of thermal, nuclear, wind, and other renewable energy technologies into hybrid power systems, addressing two major challenges: economic viability and environmental sustainability. It highlights the need for optimized scheduling and safety mechanisms in nuclear energy integration while proposing advanced machine learning-based control strategies for managing the intermittency of wind energy.
4. Real-World Validation Through Case Studies: To reinforce the practical applicability of the proposed methodologies, the study conducts real-world case studies involving FOPID controllers optimized using Firefly Optimization (FFO) and PID controllers optimized with PSO. These case studies validate the superiority of fractional-order controllers in mitigating disturbances under random loading conditions and weather variations, demonstrating their potential for improving frequency stability and overall power system resilience.
5. Resilience and Real-Time Adaptability in Power Systems: The study emphasizes the need for real-time data-driven decision-making in hybrid power systems. It explores the application of multi-agent systems enhanced by deep reinforcement learning (DRL) to handle decentralized energy networks, ensuring real-time adaptability to dynamic energy demands and operational uncertainties.
6. Cyber-Physical Security and Grid Reliability: With increasing cybersecurity threats to power grids, the research highlights the importance of integrating cybersecurity measures within optimization frameworks to safeguard power infrastructure. Additionally, the study explores how distributed energy resources (DERs), such as battery storage and smart grids, can enhance grid flexibility and resilience.
7. Hybrid Optimization Techniques for Future Power Grids: The study advocates for the development of hybrid optimization techniques that combine machine learning with traditional optimization methods to improve computational efficiency and predictive accuracy. These approaches are particularly beneficial for evolving energy systems, such as microgrids and smart grids, where rapid adaptability is essential for maintaining grid stability and operational efficiency.
8. Policy and Implementation Insights for India and Africa: Given the unique challenges faced by emerging economies, this study provides strategic insights for policymakers and grid operators in India and Africa. It emphasizes the importance of integrating scalable, cost-effective, and resilient renewable energy solutions to enhance long-term energy security, economic growth, and environmental sustainability.

### Green energy

**Fig. 2:**
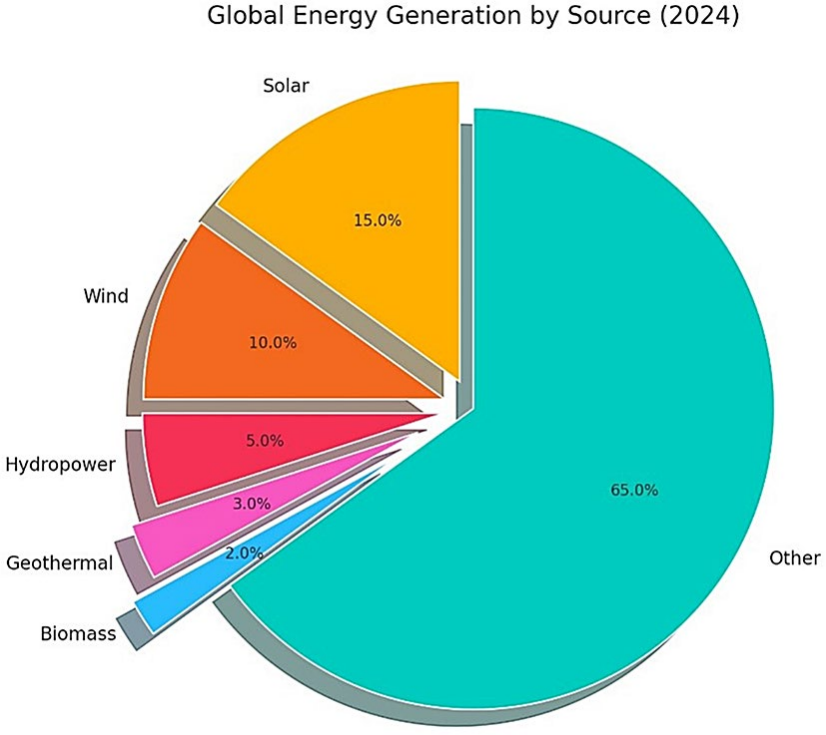
Global green energy generation (2024).

Green energy, also termed renewable or sustainable energy, encompasses power generated from naturally replenishing resources with a low environmental footprint. In contrast to fossil fuels, these energy sources emit negligible greenhouse gases and pollutants, making them vital for combating climate change and mitigating ecological harm. The global energy mix has progressively shifted toward renewables, with green energy sources comprising around 30% of total electricity generation in 2024, as illustrated in Fig. 2. This transition is fueled by the widespread implementation of various renewable technologies, further outlined below^7^.

#### Solar power

Solar power harnesses energy from the sun through two main technologies:

- **Photovoltaic (PV) Cells:** PV cells transform sunlight into electricity and are widely used in solar panels installed on roof of buildings, in solar parks, and in smaller, portable applications.
- **Solar Thermal Systems:** These systems harness solar energy by heating a working fluid, which subsequently generates steam to drive a turbine for electricity production. Concentrated Solar Power (CSP) plants are a prime example of this approach, utilizing mirrors or lenses to focus sunlight onto a targeted area, thereby achieving the high temperatures necessary for efficient thermal-to-electric energy conversion.

As global energy use increases, so do environmental concerns, making solar power a smart and clean solution. Solar power is a rapidly growing segment of the energy market, contributing a significant portion to the global renewable energy capacity. In 2024, solar energy represents a major part of the renewable mix, reflecting its accessibility and substantial cost reductions in recent years. The study in^8^ gives an overview of maximum power point tracking (MPPT) techniques to get the most energy from solar panels, even in conditions like partial shading.

#### Wind power

Wind power generates electricity using wind turbines, which are typically installed in large wind farms on land or offshore. Wind energy, contributing prominently alongside solar, is one of the most cost-effective green energy sources. Its small land footprint allows for dual land use, particularly in agricultural settings. The main components of a wind turbine include the rotor blades, nacelle (which houses the generator), and a tower.

In 2024, wind power continues to be a crucial player in the global energy mix, significantly contributing to the overall renewable energy generation as indicated in Fig. 2. Together with solar, wind energy is responsible for a substantial portion of the new renewable capacity added globally.

#### Hydro power

Hydropower, or hydroelectric power, captures the energy of flowing water. The predominant method involves constructing dams to form reservoirs, from which controlled water release flows through turbines to generate electricity. In contrast, smaller-scale systems, like run-of-river configurations, channel a fraction of the river’s flow through a turbine, producing power with minimal impact on the river’s natural course.

Hydropower remains a reliable and consistent source of energy, providing base-load power. In 2024, it continues to hold a significant share of the renewable energy mix, particularly in regions with abundant water resources, as shown in Fig. 2.

#### Geothermal energy

Geothermal energy, recognized for its sustainability and minimal carbon footprint, harnesses the thermal energy stored beneath the Earth’s surface, offering diverse applications for power generation, direct heating, and climate control in buildings. In power generation, advanced geothermal plants extract heat from underground reservoirs of hot water or steam to generate electricity. These plants are primarily categorized into three types: dry steam plants, which use geothermal steam directly to drive turbines; flash steam plants, which convert high-pressure hot water into steam via rapid depressurization; and binary cycle plants, which employ a secondary working fluid with a lower boiling point than water, making them effective even at moderate temperatures. Binary cycle systems are particularly suitable for tapping into low-to-moderate temperature geothermal resources, making them ideal for regions like parts of India and Africa, where such resources are abundant.

Beyond electricity generation, geothermal energy is applied directly in various sectors, such as district heating, greenhouse farming, aquaculture, and industrial drying. These applications exploit geothermal resources with lower temperature gradients, offering an environmentally friendly alternative to conventional fossil fuels. Additionally, geothermal heat pump systems, which utilize the relatively constant temperatures near the Earth’s surface, provide energy-efficient heating and cooling solutions for buildings. By optimizing thermal exchange, ground-source heat pumps enhance operational efficiency while reducing environmental impacts.

Advancements in drilling technologies, reservoir management, and heat extraction techniques continue to increase the efficiency and scalability of geothermal energy systems. For regions like India and Africa, where energy demand is growing rapidly, geothermal energy presents an opportunity to diversify the energy mix, improve energy access, and contribute to decarbonization goals. While geothermal energy accounts for a smaller share of global renewable energy generation as of 2024, it remains a crucial element in the transition to sustainable energy systems, particularly for providing reliable, base-load electricity with minimal environmental impact, as shown in Fig. 2.

#### Biomass

Biomass energy, derived from organic materials such as wood, agricultural residues, and animal waste, represents a sustainable and renewable resource for power generation. Its potential in advancing renewable energy systems is significant, particularly for emerging markets in India and Africa, where biomass offers an opportunity to meet increasing energy demands while contributing to environmental sustainability. Biomass can be utilized through various advanced conversion processes, each tailored to specific applications. Combustion, one of the most common methods, involves the direct burning of organic materials to generate heat or electricity. This process harnesses the chemical energy stored in biomass, converting it into thermal energy for industrial heating or power generation. Advanced combustion technologies, such as fluidized bed and gasification systems, improve efficiency by optimizing combustion processes and reducing harmful emissions. These systems are increasingly implemented in both developed and developing regions, including India and Africa, where biomass resources are abundant but underutilized.

Anaerobic digestion is another key process in biomass energy production, involving the breakdown of organic matter in the absence of oxygen, leading to the generation of biogas. The biogas, mainly composed of methane, can be used for electricity generation, heating, or as a renewable fuel for transportation. This process is particularly valuable in waste-to-energy applications, as it contributes both to energy production and waste management. In regions like India and Africa, where agricultural and animal waste are abundant, anaerobic digestion provides a cost-effective solution for waste disposal and energy generation. Additionally, biomass can be converted into liquid biofuels such as ethanol and biodiesel through processes like fermentation and transesterification. These biofuels are increasingly used as substitutes for conventional fossil fuels in transportation, reducing greenhouse gas emissions. Ongoing research and development efforts are focused on improving biofuel production efficiency, particularly through second- and third-generation feedstocks, which utilize non-food biomass and waste materials. This development is particularly relevant for countries like India and those in Africa, where large agricultural sectors can contribute to sustainable biofuel production.

The diverse biomass conversion processes play a critical role in the transition to sustainable energy systems, contributing to carbon emission reduction, enhanced energy security, and sustainable resource management. Biomass is considered CO₂-neutral, as the carbon dioxide released during combustion is largely offset by the carbon absorbed by plants during their growth cycle, creating an approximate equilibrium in carbon emissions over the biomass lifecycle. For nations in India and Africa, which face energy access and environmental challenges, biomass energy offers a dual advantage: addressing energy shortages while mitigating climate change impacts. Despite its relatively modest share in the renewable energy portfolio as of 2024, biomass energy continues to hold significant importance in specific regions, as illustrated in Fig. 2.

#### Nuclear energy

Nuclear energy occupies a unique position in the green energy debate because it can produce substantial electricity output while emitting very low levels of greenhouse gases during operation. While it shares some characteristics with renewable energy sources, it also presents distinct challenges that need to be addressed.

Nuclear energy is primarily produced through nuclear fission, a process in which the nucleus of an atom (usually uranium-235 or plutonium-239) splits into two smaller nuclei, along with a few neutrons and a large amount of energy. This energy is used to heat water into steam, which drives turbines connected to generators to produce electricity. There is also ongoing research into nuclear fusion, which powers the sun and stars, but practical and commercial fusion reactors remain a future goal.

- **Low Greenhouse Gas Emissions**: Nuclear power units emit virtually no direct greenhouse gases during operation, significantly reducing their impact on climate change compared to fossil fuel-based power plants. Over its entire lifecycle, including construction, operation, and decommissioning, the carbon footprint of nuclear energy is comparable to that of wind and solar energy.
- **Increased Energy Concentration**: Nuclear energy has a much-increased energy density than fossil fuels and renewables. A small amount of nuclear fuel can produce a vast amount of energy, reducing the need for extensive fuel extraction, processing, and transportation, which further minimizes environmental impacts.

Despite these benefits, nuclear energy contributes to only a modest share of the global energy mix in 2024 as represented in Fig. 2. However, its potential for providing reliable, base-load power makes it a critical component of a balanced energy portfolio.

Nuclear energy faces several challenges and concerns that must be addressed to ensure its safe and sustainable use.

- **Radioactive Waste**: One of the most significant challenges of nuclear energy is the production of radioactive waste, which requires secure and stable storage solutions.
- **Nuclear Accidents**: Accidents at nuclear power plants, though rare, can have severe and long-lasting environmental and health consequences.
- **Nuclear Proliferation**: The dual-use nature of nuclear technology—capable of serving both civilian energy needs and military applications—raises significant concerns about nuclear proliferation, particularly in the context of global security and regional stability. In India, this dual-use potential is especially pertinent. While nuclear energy offers a pathway to curb dependency on geological fuels and support the nation’s growing energy demands sustainably, it also brings the challenge of strict oversight and control to prevent any diversion of nuclear materials toward weaponization. As a nuclear-armed state and a responsible member of the global non-proliferation framework, India faces the unique challenge of balancing its nuclear energy ambitions with commitments to prevent the spread of nuclear weapons technology. This balance necessitates advanced safeguards, transparent policies, and international cooperation to ensure that the peaceful applications of nuclear technology do not inadvertently contribute to nuclear risks in the region.
- **Cost**: The high upfront capital investment and long lead times required for nuclear power plants present significant financial challenges.

Despite these challenges, advances in nuclear technology offer promising solutions:

- **Generation IV Reactors**: These advanced reactors are optimized for improved safety, performance, and produce less waste than current reactors.
- **Small Modular Reactors (SMRs)**: SMRs offer a scalable and potentially safer nuclear option, with lower upfront costs and shorter construction times.
- **Fusion Power**: Though still in the research phase, nuclear fusion holds the promise of a nearly limitless supply of energy with minimal waste.

The inclusion of green energy into the Indian and African grid systems represents a multifaceted yet transformative challenge, balancing the urgent demand for reliable and sustainable energy with the technical intricacies of renewable energy deployment. In India, rapid economic expansion and a burgeoning population have significantly escalated energy demands, driving a strategic pivot toward green energy resources i.e. solar, wind, hydro, and biomass. Solar energy, in particular, assumes a pivotal role due to India’s abundant solar insolation. Flagship programs like the National Solar Mission Strive to position the nation as a global frontrunner in solar energy production. This vision is being realized through the proliferation of utility-scale solar parks and rooftop photovoltaic (PV) systems, underpinned by plummeting PV technology costs and robust policy incentives.

Despite these advancements, integrating solar power into the national grid presents substantial challenges. The inherent intermittency of solar energy demands sophisticated grid management strategies to maintain stability and reliability. Advanced energy storage technologies, such as lithium-ion batteries, flow batteries, and pumped hydro storage, are being deployed to mitigate variability and ensure seamless power delivery. Additionally, smart grid innovations, real-time monitoring systems, and predictive analytics are being explored to enhance grid resilience and optimize renewable energy integration.

Similarly, in African grid systems, the potential for green energy utilization is immense, given the continent’s rich renewable energy resources. However, challenges such as underdeveloped infrastructure, limited financial resources, and technical expertise require targeted interventions. Both regions exemplify the critical need for scalable, adaptive solutions that combine policy support, technological innovation, and international collaboration to transition toward sustainable and resilient energy systems.

Wind energy is another key resource, particularly along India’s coastal states like Tamil Nadu and Gujarat, where favourable wind speeds provide substantial generation potential. The integration of wind power, however, demands high-capacity transmission lines to move energy from wind-abundant regions to major load centers, requiring robust grid infrastructure investments to maintain power quality and reliability. Hydropower has traditionally been part of India’s energy mix and is instrumental in balancing intermittent renewable sources. Biomass energy, primarily derived from agricultural residues, offers a dispatchable renewable source, with biomass gasification and waste-to-energy plants supporting grid resilience, especially in rural areas. Despite the strides India has made, challenges remain in ensuring grid reliability amid renewable fluctuations, necessitating advanced energy management and real-time analytics to optimize demand-response capabilities. Additionally, India is exploring green hydrogen as an emerging energy storage solution for seasonal energy balancing, with potential applications in industrial decarbonization.

In Africa, the grid system faces distinct challenges and opportunities shaped by the continent’s vast renewable resources and the urgent need for expanded electrification. With over 600 million people still lacking access to reliable electricity, green energy holds transformative potential for Africa’s socio-economic development. Solar energy is a key focus area, particularly in the Sahel and northern African regions, where abundant sunlight makes solar power a feasible solution for electrification. Countries like Egypt, Morocco, & South Africa Have committed substantial resources to large-scale solar initiatives, using both concentrated solar power (CSP) and PV technologies. Off-grid solar systems are also critical in Africa, offering decentralized solutions for communities disconnected from national grids. Innovative financing models, such as pay-as-you-go solar, have made it possible for rural households to access clean energy affordably, contributing significantly to the continent’s electrification goals.

Wind energy, particularly in Africa’s coastal regions and highlands, is another promising area. Projects such as Kenya’s Lake Turkana Wind Power project and Senegal’s Taiba N’Diaye Wind Farm underscore the potential of wind energy for grid-scale applications. However, grid integration of wind energy remains technically challenging due to limited transmission infrastructure. Initiatives such as the East African Power Pool (EAPP) and the West African Power Pool (WAPP) aim to address this by creating cross-border interconnections to facilitate energy sharing and stabilize the grid. Hydropower also features prominently in African renewable energy strategies, especially in central and eastern regions. Major projects like Ethiopia’s Grand Renaissance Dam provide reliable, renewable power while also offering water management capabilities that benefit agricultural and urban areas alike. In East Africa’s Rift Valley, geothermal resources are being tapped, with Kenya and Ethiopia leading in geothermal development. Geothermal energy provides a stable, base-load source that complements variable green resources like solar and wind, thereby enhancing grid stability.

Despite these advances, Africa’s power grid infrastructure faces significant hurdles, including frequent blackouts and voltage instability, exacerbated by the intermittency of renewable sources. Microgrids and hybrid renewable systems, particularly solar PV combined with battery storage, are gaining traction as a solution for delivering reliable electricity to off-grid and remote areas, reducing dependency on diesel generators. Additionally, many African countries are adopting policies that encourage renewable energy investments, supported by the African Union and international development agencies. These efforts include capacity-building initiatives to develop technical skills for maintaining and expanding renewable energy infrastructure sustainably.

The renewable energy initiatives of India and Africa illustrate two distinct yet complementary approaches to green energy integration within grid systems. Both regions are investing in diverse renewable sources, grid infrastructure upgrades, and advanced storage and management solutions to address the variability of renewable energy. These strategies support energy independence and carbon reduction goals, and, through continued investment, policy support, and international collaboration, India and Africa are positioned to achieve sustainable, resilient energy systems that foster economic growth and environmental sustainability.

#### Role in a green energy future

Nuclear energy can play a complementary role alongside renewable energy sources in achieving a green energy future. Its ability to provide reliable, base-load power makes it an attractive option for maintaining grid stability, particularly in regions where renewable resources are limited or inconsistent. However, the deployment of nuclear energy must be balanced with careful consideration of safety, waste management, and non-proliferation concerns. In 2024, the global energy generation mix, as shown in Fig. 2, is defined by a growing share of renewables, including solar, wind, hydropower, geothermal, and biomass, which together account for about 30% of the total electricity generated. Nuclear energy, while a smaller part of the mix, continues to play a vital role in providing large-scale, low-emission power. By addressing the associated challenges through technological advancements and stringent regulatory frameworks, nuclear energy can contribute to a sustainable and reliable energy future, complementing renewable sources to reduce reliance on fossil fuels and combat climate change.

Transitioning from the discussion of green power sources and their influence on the international energy composition, it is equally important to consider how the power extracted from these and other sources is effectively managed and delivered to meet consumer demands. This involves not only inclusion of green energy into the grid but also the sophisticated control mechanisms required to maintain the stability and reliability of the power system. One critical aspect of this management is Generation Control.

### Generation control

Generation control refers to the methods and strategies used to manage and regulate the output of power yielding units. The primary objectives are to ensure a continuous supply of electricity that meets the demand, maintains voltage levels within desired ranges, and optimizes the operation of power plants for efficiency and cost-effectiveness. The major components of generation control include:

- **Economic Dispatch**: This involves determining the most cost-effective combination of power generation units to meet the required load while minimizing fuel costs and adhering to operational constraints. Economic dispatch algorithms consider factors such as fuel prices, generation unit efficiencies, and operational limits.
- **Unit Commitment**: This refers to the scheduling of power generation units to ensure that sufficient capacity is available to meet anticipated load demands. Unit commitment decisions take into account factors such as startup and shutdown costs, minimum up and down times, and system reliability requirements.
- **Automatic Generation Control (AGC)**: AGC is a system that automatically adjusts the output of multiple generators to match load demand in real-time. It helps maintain system frequency and tie-line power flows within specified limits. AGC uses signals from the system operator to adjust the generation output based on deviations from the scheduled values.

Following the principles of Generation Control, which focus on managing and optimizing the output of power generation units, it is essential to also address the dynamic challenges associated with maintaining the system’s overall frequency stability. This is where Load Frequency Control (LFC) comes into play, ensuring that the power grid remains stable by precisely balancing generation with load demand in real-time.

#### Load frequency control (LFC)

LFC is a vital function in power system operations, focusing on sustaining system frequency within specified limits and upholding grid stability. Effective frequency control is essential, as system frequency reflects the equilibrium between power generation and demand; any discrepancy can result in frequency deviations, potentially causing equipment damage, grid instability, or even widespread blackouts. Key components of LFC include:

- **Primary Frequency Control**: Also known as droop control, primary frequency control is the initial response to frequency deviations. It involves the automatic adjustment of generator output in response to changes in system frequency. Generators are equipped with governors that sense frequency changes and adjust the mechanical power input to the turbines accordingly.
- **Secondary Frequency Control**: Also known as AGC, secondary frequency control fine-tunes the response initiated by primary control. It operates over a longer time scale (minutes) and adjusts the generation output to bring the frequency back to its nominal value and restore any deviations in tie-line power flows between interconnected areas.
- **Tertiary Frequency Control**: This involves manual or automated adjustments to generation dispatch and load shedding to restore the balance between supply and demand. Tertiary control is typically used for longer-term frequency regulation and is activated in response to significant disturbances or to relieve primary and secondary control resources.

#### Interaction between generation control and LFC

Generation control and LFC are closely intertwined in the operation of power systems. Effective generation control ensures that there is sufficient and cost-effective generation capacity available to meet load demands, while LFC maintains system frequency and stability by continuously adjusting generation output in response to load changes.

The interaction between these controls is essential for:

- **Maintaining System Reliability**: Ensuring that the power system operates within safe and stable frequency and voltage limits.
- **Economic Efficiency**: Minimizing operational costs by optimizing the use of generation resources.
- **System Resilience**: Enhancing the ability of the power system to withstand and recover from disturbances.

In today’s evolving power systems, the incorporation of advanced strategic technologies such as real-time surveillance, predictive analytics, and machine learning has become essential to enhancing generation control and LFC capabilities. These technologies play a crucial role in maintaining system resilience and performance, especially as electrical grids become increasingly complex due to the rise of green energy penetration. Generation control and LFC mechanisms work to balance power generation with demand fluctuations, ensuring system frequency remains within specified limits and thus providing consumers with reliable, cost-effective power.

In India and Africa, where renewable energy integration is expanding rapidly, the role of generation control and LFC is becoming even more critical. In India, high solar and wind energy generation, coupled with the growing share of grid-connected renewable sources, demands adaptive generation control systems to manage the intermittency of these resources. Real-time monitoring and predictive analytics enable India’s grid operators to anticipate fluctuations in renewable output and respond dynamically, helping to maintain frequency stability and minimize disruptions. Machine learning algorithms are also being used to optimize load forecasting and predict generation patterns based on weather data, making it possible to better match supply with demand even under variable conditions. With these advancements, India’s grid can enhance both resilience and efficiency, supporting the national goal of achieving substantial renewable energy penetration in the coming decades.

In Africa, where energy access and grid reliability are pressing issues, advanced generation control and LFC are essential for integrating decentralized green energy strategies such as off-grid solar and microgrids. As several African countries invest in regional power pools and cross-border interconnections, coordinated LFC becomes vital for managing frequency stability across interconnected grids. Predictive analytics help in pre-emptively identifying and addressing potential instabilities caused by load variations, which are common in areas with underdeveloped grid infrastructure. Machine learning-based optimization algorithms also aid in managing distributed energy resources, enabling smoother integration of renewables into the national grids while mitigating the frequency instability often seen in weak grids.

The impact of these advanced optimization techniques extends beyond mere operational efficiency. By improving generation control and LFC, both India and Africa are able to reduce their dependency on fossil fuel-based power plants during peak loads, lowering emissions and aligning with international climate goals. Furthermore, improved grid stability encourages private investments in renewable projects, as energy developers are more assured of grid reliability. This, in turn, accelerates renewable energy growth and drives economic development through job creation in both regions.

As renewable energy penetration continues to rise in these regions, the optimization of generation control and LFC will remain a cornerstone of energy security and sustainability. By leveraging data-driven approaches and cutting-edge technologies, India and Africa can ensure their power systems are both resilient and prepared to meet the challenges of a green energy future. Through the integration of these advanced solutions, they will foster robust, sustainable, and economically viable power systems that support broader social and environmental progress.

The evolution of LFC in interconnected power systems has been a pivotal area of research for decades. Initial contributions laid the foundation for frequency stability in interconnected networks^9,10^. Early studies proposed continuous and tie-line control mechanisms for frequency regulation in multi-area systems^11,12^, while innovative techniques like load-phase and constant-frequency control began to emerge in the mid-twentieth century^13,14^.

By the late 1960 s, the interrelation of time error, frequency deviation, and inadvertent flows was highlighted^15^, and large signal dynamics were modeled using nonlinear optimization methods^16^. Further advancements considered the plant response effect on optimal LFC design^17^and the incorporation of digital control in automatic generation control (AGC) systems^18^.

Decentralized and optimal control frameworks gained momentum in the 1970 s, introducing dynamic programming and nonlinear control strategies^19–21^. The impact of renewable energy sources such as wind was first examined in the early 1980 s^22^, while decentralized optimization methods continued to be refined^23^. Real-time optimal power flow integration into AGC emerged in the late 1980 s^24^, supported by generalized and predictive algorithms^25,26^.

The 1990 s witnessed the emergence of real-time pricing strategies^27^, discrete-mode control models^28^, and AC/DC tie-line coordination^29^. Various advanced control strategies, including integral control, three-level control schemes, and integrator decoupling methods, were also introduced^30–34^. Researchers began addressing challenges with non-conforming loads^35^and automatic control for multi-area systems^36^.

With power system deregulation, new control paradigms were developed to adapt to market-driven environments^37,38^. The early 2000 s saw the rise of intelligent control techniques such as fuzzy logic^39–42^, extended integral controllers^43^, and hybrid system modeling with conventional controllers^44^.

As power systems became more complex, LFC research expanded to consider multi-area restructured systems^45^, evolutionary optimization techniques^46^, and robustness under uncertainty using adaptive PID tuning^47^. The integration of metaheuristics like craziness-based particle swarm optimization^48^, and hybrid control elements such as SMES-TCPS combinations^49^, marked a shift towards more intelligent and adaptive solutions.

Recent developments focus on multi-source power systems, combining conventional, renewable, and hybrid energy systems with optimization-based control strategies^50,51^. These studies emphasize the role of soft computing techniques in improving frequency stability, dynamic response, and operational flexibility of modern interconnected grids.

### Optimization

Optimization is a core concept across multiple area, including mathematics, technology, finance, and informatics practices. It entails identifying the most optimal solution from a set of feasible alternatives to achieve a defined objective, which may involve maximizing or minimizing factors like profit, cost, efficiency, or performance. In decision-making scenarios where resources are constrained, optimization is essential for ensuring their efficient allocation.

Types of optimizations play a crucial role in various decision-making processes by offering tailored approaches to different types of problems. These methods ensure that specific objectives, whether they involve maximizing efficiency or minimizing costs, are met effectively. It involves finding the best solution from a set of feasible solutions to achieve a specific objective. This objective could be maximizing or minimizing a particular function, such as profit, cost, efficiency, or performance. Optimization plays a crucial role in decision-making processes where resources are limited and must be allocated efficiently.

Optimization is a cornerstone of decision-making and system design, addressing the challenge of identifying the best solution under defined constraints. Linear optimization, or linear programming, focuses on optimizing a linear objective function subject to a set of linear equality or inequality constraints. Widely used in industries such as resource allocation, production scheduling, transportation, and logistics, it relies on robust algorithms like the simplex method and interior-point methods, with recent advancements enabling scalability to large-scale problems. Nonlinear optimization, on the other hand, is crucial when the objective function or constraints are nonlinear. These inherently complex problems are tackled using advanced techniques such as gradient descent, Newton’s method, and conjugate gradient methods, finding applications in fields like machine learning, financial modeling, and engineering design, where precision and adaptability are essential.

Integer optimization addresses scenarios where decision variables are constrained to integer values, making it indispensable for problems like scheduling, network design, and resource allocation. This domain employs methods such as branch-and-bound and cutting-plane algorithms to find optimal solutions. Mixed-integer optimization, which combines integer and continuous variables, is particularly suited for hybrid decision-making tasks, including supply chain management, energy system optimization, and telecommunications. For problems requiring optimal combinations from finite sets, combinatorial optimization offers robust solutions through techniques like dynamic programming, greedy algorithms, and heuristic approaches, solving classic problems such as the traveling salesman problem and the knapsack problem.

In dynamic environments with inherent uncertainties, stochastic optimization becomes vital. By modeling uncertain parameters as random variables, it enables robust decision-making using methods like Monte Carlo simulation, stochastic gradient descent, and robust optimization. This approach finds significant applications in financial portfolio management, inventory control, and uncertainty-resilient decision-making. Meanwhile, multi-objective optimization addresses real-world problems involving conflicting objectives, such as maximizing profit while minimizing environmental impact. Pareto optimization is a prominent technique that identifies trade-offs, resulting in Pareto-optimal solutions where no objective can improve without compromising another.

Advanced methodologies further enhance the optimization landscape. Gradient-based methods, including gradient descent and quasi-Newton approaches, are efficient for navigating large-scale problems but may face challenges with non-convex functions. Evolutionary algorithms, inspired by natural selection, such as genetic algorithms, differential evolution, and particle swarm optimization, provide robust solutions for non-linear and multi-modal landscapes, albeit with computational intensity requiring parallel computing. Simulated annealing, which mimics the metallurgical annealing process, excels in escaping local optima, making it suitable for highly complex problems, though it requires careful parameter tuning for efficiency.

Heuristic and metaheuristic approaches, such as greedy algorithms, tabu search, and ant colony optimization, offer practical solutions for computationally infeasible problems, especially in real-time scenarios. Constraint programming, which declaratively specifies constraints, is highly effective for combinatorial and scheduling challenges. Machine learning-based optimization leverages data-driven models, including neural networks and reinforcement learning, to solve dynamic and evolving problems with adaptive, scalable solutions.

This taxonomy of optimization techniques underscores their critical role in solving complex problems across diverse domains. As computational advancements and algorithmic innovations continue, these methodologies are poised to drive breakthroughs in efficiency, precision, and scalability, reshaping optimization-driven decision-making and enabling transformative solutions for the future.

The versatility and power of optimization techniques make them applicable across a wide range of industries and sectors. These techniques play a crucial role in improving efficiency, reducing costs, and enhancing decision-making processes. Below are some key applications of optimization that demonstrate its impact in various fields.

- **Industrial and Manufacturing**: Optimization is used to design efficient production processes, minimize waste, schedule maintenance, and manage supply chains.
- **Finance**: Portfolio optimization, risk management, option pricing, and algorithmic trading are key areas where optimization techniques are applied.
- **Transportation and Logistics**: Route planning, vehicle scheduling, fleet management, and network optimization are critical for reducing costs and improving service levels.
- **Energy Systems**: Optimization helps in the efficient operation of power grids, integration of renewable energy sources, and energy storage management.
- **Healthcare**: Resource allocation, scheduling of medical staff, treatment planning, and drug formulation are areas where optimization can lead to better outcomes and cost savings.
- **Telecommunications**: Network design, bandwidth allocation, and traffic management are optimized to ensure reliable and efficient communication services.
- **Environmental Management**: Optimization is used to balance economic and environmental objectives, such as in pollution control, resource management, and sustainable development planning.

Optimization is a powerful and versatile tool that plays a crucial role in improving efficiency, reducing costs, and enhancing decision-making across various domains. As computational techniques and algorithms continue to advance, the ability to solve increasingly complex and large-scale optimization problems has grown, enabling more effective and innovative solutions to real-world challenges. However, many real-world problems are so complex that finding the exact optimal solution can be extremely difficult, if not impossible, within a reasonable timeframe. This is where heuristics and metaheuristics become invaluable.

#### Heuristics

Heuristic methods are practical approaches used to find satisfactory solutions to optimization problems, particularly when finding the exact optimal solution is computationally impractical or impossible. These methods leverage experience, rules of thumb, and educated guesses to quickly arrive at effective solutions, even if they do not guarantee the globally optimal outcome. Heuristics employ a variety of strategies to address complex problems where the search for an exact solution may be infeasible. Among these strategies, several distinct approaches are commonly utilized, each offering specific strengths and applications.

Greedy algorithms are a classic heuristic approach that makes step-by-step decisions, selecting the best available option at each stage with the expectation of finding a global optimum. For example, the greedy algorithm applied to the knapsack problem and Dijkstra’s algorithm for shortest path problems both utilize this decision-making process. Although greedy algorithms are efficient and easy to implement, they often fail to find the globally optimal solution due to their focus on local optimization. Local search methods, such as hill climbing and simulated annealing, begin with an initial solution and iteratively improve it by exploring neighbouring solutions with better objective function values. These methods are simple, effective, and widely used, but they may get stuck in local optima, especially in high-dimensional, multimodal search spaces. Constructive heuristics, such as the nearest neighbour algorithm for the traveling salesman problem (TSP), build a solution incrementally by adding one element at a time, often yielding fast and reasonable solutions, though they may not be optimal.

Metaheuristics, in contrast, provide higher-level frameworks that guide heuristics in the search for solutions, offering a more structured approach to problem-solving. Metaheuristics are designed to escape local optima and explore the solution space more effectively, making them particularly valuable for complex optimization problems where traditional methods may fail. These techniques aim to enhance the search process by allowing more extensive exploration of the solution space while avoiding premature convergence to suboptimal solutions.

Genetic algorithms (GA), inspired by the concept of natural selection, evolve a population of candidate solutions over multiple generations through mechanisms such as selection, crossover, and mutation. These algorithms are highly adaptable and robust, making them effective for a broad spectrum of optimization problems, including scheduling, design, and machine learning. Simulated annealing (SA), a probabilistic optimization technique, mimics the metallurgical annealing process, allowing for occasional uphill moves to escape local optima. Over time, the probability of these uphill moves decreases, guiding the algorithm toward the optimal solution. Although SA is effective for large and complex problems, it can be computationally intensive and may converge slowly. PSO, inspired by the collective behavior of birds and fish, iteratively refines a population of candidate solutions (particles) by adjusting their positions and velocities within the search space^52^. PSO has been particularly successful in solving high-dimensional continuous optimization problems.

Ant colony optimization (ACO), drawing from the foraging behavior of ants, uses pheromone trails to direct the search for optimal solutions. This approach has shown particular effectiveness in solving combinatorial optimization problems, such as the traveling salesman problem and network routing^53^. Tabu search enhances local search techniques by maintaining a memory structure, known as a tabu list, which records previously visited solutions. This prevents revisiting them and allows the algorithm to explore new regions of the search space, thus avoiding local optima^54^. Differential evolution (DE) optimizes solutions by refining a population of candidate solutions based on the differences between randomly selected solution pairs. This iterative approach enables DE to explore the solution space efficiently and converge to optimal solutions, especially in continuous optimization scenarios^55^. Lastly, harmony search (HS), inspired by the improvisational techniques of musicians, explores the solution space by utilizing a harmony memory, which stores the best discovered solutions. New solutions are generated by blending elements from these stored solutions, iterating toward the optimal outcome^56^. The system incorporating nonlinearities was analyzed and effectively addressed using ABC algorithm by the authors in^57^. Additionally, advancements in frequency stability through fuzzy-based techniques have been explored in^58^.

In conclusion, both heuristic and metaheuristic techniques play crucial roles in solving complex optimization problems, particularly when traditional methods are impractical. Heuristics offer fast, satisfactory solutions, while metaheuristics provide more structured frameworks to guide heuristics, ensuring effective exploration of the solution space and avoiding local optima. These advanced optimization methods have been successfully applied across numerous domains, including engineering design, scheduling, and machine learning, demonstrating their efficiency and adaptability in tackling challenging optimization tasks.

Heuristics and metaheuristics offer powerful tools for tackling complex optimization problems where traditional methods may be inadequate. By combining practical rules with sophisticated search strategies, these techniques can deliver good solutions efficiently, making them invaluable in various fields ranging from engineering to finance. As computational power continues to advance, the role of heuristics and metaheuristics in solving real-world problems will likely expand, opening new possibilities for optimization in increasingly complex environments^55^.

## Literature survey

### Transition to green energy

The transition to green energy is a crucial component in the global effort to address climate change and foster sustainable development. Numerous studies have examined the integration of renewable energy sources, regulatory frameworks, and the contribution of nuclear energy to these objectives. Aydin et al.^59^ emphasize the necessity for comprehensive regulatory frameworks that integrate socio-economic elements such as human rights, banking sector development, and economic freedom. Their research underscores the importance of regulatory strategies that facilitate sustainable environmental management, particularly in the context of green energy transitions. Similarly, Shi et al.^60^ investigate the efficiency of low-carbon and high-ratio green energy systems, focusing on advanced control strategies within Combined Cooling, Heating, and Power (CCHP) systems. Their findings highlight the potential for substantial improvements in system efficiency and carbon emission reductions through optimized control mechanisms.

Nuclear energy’s role in promoting both economic growth and environmental sustainability is another central theme in the literature. Teng et al.^61^ find a positive correlation between nuclear energy usage and improved load capacity factors and economic progress in leading nuclear power economies, highlighting its dual benefits. Kirikkaleli et al.^62^ further demonstrate that nuclear energy consumption significantly contributes to economic growth in the UK, while Danish et al.^63^ confirm that nuclear energy plays a pivotal role in reducing CO2 emissions in India, supporting the IPAT and EKC hypotheses. These conclusions are echoed by Kim^64^, who stresses the importance of investing in both nuclear and renewable energy to reduce greenhouse gas emissions in Korea. Luqman et al.^65^ show that both nuclear and renewable energy sources positively impact economic growth in Pakistan, with nuclear energy having a notably stronger effect. Saidi et al.^66^ build on this by illustrating that the integration of these energy sources effectively reduces CO2 emissions in OECD countries. Sarkodie et al.^67^ further emphasize that the environmental benefits of nuclear and renewable energy are maximized when backed by strong political institutions. Complementing these findings, Mahmood et al.^68^ demonstrate that nuclear energy is key in reducing environmental pollution in Pakistan, underscoring its significance in sustainable development efforts.

Regarding clean energy transitions, Imran et al.^69^ and Yue et al.^70^ explore the financial and environmental impacts of nuclear energy, arguing that it is essential for reducing carbon emissions and promoting sustainable development, particularly when coupled with stringent environmental policies and innovative financial mechanisms like carbon taxes. Matsui et al.^71^ and Huang et al.^72^ add that greater investment in nuclear energy technology leads to substantial reductions in CO2 emissions, reinforcing its role in global climate strategies. Zheng et al.^73^ highlight the importance of nuclear energy and robust environmental policies in achieving sustainable development targets. Finally, Aktekin et al.^74^ assess the techno-economic viability of hybrid systems that integrate nuclear and renewable energy, concluding that such systems offer a sustainable and economically feasible solution for future energy demands.

Overall, the literature highlights the multifaceted role of nuclear energy in facilitating green energy transitions, driving economic growth, and ensuring environmental sustainability. Effective regulatory frameworks, advanced control strategies, and significant financial investments are identified as essential components for optimizing the benefits of both nuclear and renewable energy sources. Comprehensive policies and technological advancements are critical to achieving global sustainability objectives and addressing the challenges posed by climate change.

#### Impact of climate change on power systems

The increasing unpredictability of power systems due to climate change, particularly concerning solar energy generation and weather-sensitive loads, presents significant challenges for electricity planning and grid stability. To address this, the study in^75^ employs generative adversarial networks (GANs) to generate realistic weather data and assess its impact on electricity distribution. By improving the accuracy of weather-dependent power forecasts, this approach enhances grid planning and operational efficiency. Similarly, research in^76^ utilizes GAN-based models to refine the prediction of solar power fluctuations over time. More precise forecasting methodologies contribute to improved electricity market operations, ensuring a more stable and resilient future grid.

#### Optimizing agricultural microgrids for sustainable farming

Agricultural microgrids play a critical role in enhancing energy efficiency, reducing carbon emissions, and modernizing farming practices. The study in^77^ demonstrates the benefits of co-optimizing greenhouse energy consumption and microgrid operations. A real-world implementation in Qingdao, China, highlights that this strategy significantly lowers operational costs and carbon footprints in modern agriculture. Additionally, traditional rural power networks often fall short in meeting contemporary agricultural energy demands. Research in^78^ develops an integrated energy-crop growth-carbon emission optimization model, ensuring a more sustainable and cost-effective rural energy system. A case study in Hebei, China, validates this approach, demonstrating its effectiveness in reducing both energy costs and emissions.

#### Integrated energy systems for rural sustainability

The integration of renewable energy technologies with rural agricultural infrastructure is crucial for achieving carbon neutrality and sustainable development. The study in^79^ focuses on optimizing energy usage in photovoltaic (PV) greenhouses, effectively balancing agricultural and power generation needs. A simulation conducted in northern China indicates that a 3996 m^2^ greenhouse with 25% PV coverage can reduce energy costs by 15%, showcasing the potential of solar-integrated farming systems in enhancing rural energy sustainability. By leveraging advanced forecasting techniques, optimizing microgrid operations, and integrating renewable energy systems, these studies contribute to building resilient, efficient, and low-carbon power and agricultural networks in response to climate change.

#### Energy management and economic viability

Renewable energy plays a pivotal role in achieving sustainable development, particularly in emerging markets such as India and Africa, where energy access and grid stability remain critical challenges. The study in^80^ evaluates hybrid renewable energy systems, analyzing the optimal integration of solar PV, wind, hydrokinetic, battery storage, and diesel generators based on real-world data. While the research is based in southern Ecuador, its findings are highly relevant to India and African nations, where decentralized and off-grid hybrid systems are essential for rural electrification and reducing reliance on fossil fuels.

The study highlights the cost-performance trade-offs of different configurations. The most cost-effective system, integrating solar PV, wind, and hydrokinetic energy with diesel backup, achieved an electricity cost of 0.36 USD/kWh, whereas a fully renewable system (without diesel) raised costs to 0.88 USD/kWh. This cost difference underscores the current economic challenges of 100% renewable systems, especially in regions with limited grid infrastructure and fluctuating renewable generation. The research also demonstrates that optimizing battery charge limits helps lower both costs and CO₂ emissions, while load-following control strategies significantly reduce diesel fuel consumption. Emission analysis for power generation from different energy sources such as coal, natural gas, petroleum, etc. is detailed in^81,82^ and ^83^.

For India and Africa, where many remote areas rely on diesel generators due to grid limitations, these findings emphasize the importance of balanced hybrid energy systems. By leveraging tools like HOMER Pro for simulation and optimization, policymakers and energy planners can design cost-effective, low-carbon microgrid solutions that ensure both economic feasibility and long-term sustainability. The integration of solar, wind, hydro, and optimized storage solutions presents a viable path for achieving reliable and affordable clean energy access across underserved regions in these markets.

### Optimization of renewable energy systems

Recent research has made significant progress in optimizing renewable energy systems, focusing on solar, wind, and hydrogen technologies. These advancements are particularly crucial for regions like India and Africa, where integrating renewable energy is essential to meet rising energy demands and sustainability goals. Several studies have explored optimization techniques for energy storage, system integration, and green hydrogen production. Kaan et al.^84^ emphasize the importance of efficient energy storage for solar- and wind-powered green campuses, highlighting its role in improving grid resilience, especially in regions with intermittent renewable generation. Similarly, Daaboul et al.^85^ analyze the net green energy potential of solar and wind systems, stressing the need for optimized integration strategies to maximize efficiency.

Hybrid renewable energy systems are gaining traction as viable solutions for energy security. Liu et al.^86^ demonstrate that integrating hydrogen storage with wind and solar technologies enhances sustainability, a promising approach for India and Africa, where fluctuating renewable generation poses challenges. Ahmed et al.^87^ introduce triboelectric nanogenerators as an innovative wind energy harvesting method, which could complement existing technologies in remote areas with limited grid infrastructure. Further research highlights the role of regional renewable energy strategies. Hassan et al.^88^ examine the European energy landscape, offering insights into optimizing solar, wind, hydro, and hydrogen energy—lessons applicable to India and Africa. Awad et al.^89^ extend this analysis to water electrolysis for green hydrogen production, discussing hybrid approaches that could drive decarbonization. Similarly, studies on wind-hydrogen integration^90^, hydrogen production from surplus renewable energy^91^, and advanced turbine technologies^92,93^ underscore the growing role of optimization in enhancing renewable energy adoption.

In summary, optimizing renewable energy systems requires an integrated approach, combining advanced storage solutions, hybrid energy configurations, and innovative technologies. For India and Africa, these advancements are crucial to improving grid stability, ensuring energy access, and supporting the transition to a sustainable energy future. Green hydrogen, enhanced wind-solar integration, and efficient storage solutions remain key areas for further research and development.

### Generation control

The field of generation control in power systems has undergone significant transformations from 1945 to the present, driven by technological advancements and the increasing complexity of power grids.

From 1945 to 1990, initial research primarily focused on Automatic Gain Control (AGC) within single and two-area thermal systems. These early studies explored system behaviour through alterations in parameters like speed regulation, frequency bias, area capacity, and tie-line capacity. During this period, the focus was on understanding the basic dynamics of power systems under different operating conditions and how AGC could be optimized for better performance.

Between 1991 and 2010, the incorporation of optimal control theory alongside classical controllers marked a new phase in generation control. This era saw the evolution of multi-area systems and the introduction of deregulated or restructured power systems. Researchers began integrating more sophisticated control methods, such as those combining classical controllers with optimal control strategies, to manage the increasingly complex power networks more efficiently.

Since 2010, the scope of generation control in power systems has undergone significant expansion, driven by the integration of diverse energy sources such as gas, geothermal, and renewable technologies like wind and solar power. This evolution has necessitated the estimation and measurement of system parameters to address the complexity and variability introduced by renewable energy. Advanced control strategies have been developed and implemented, including integer-order (IO), fractional-order (FO), and intelligent controllers, alongside multi-degree-of-freedom (DOF) and cascade configurations such as Tilt Integral Derivative (TID) controllers. These innovations enhance the robustness and stability of power grids, ensuring reliable operation amidst the dynamic challenges posed by fluctuating power generation.

Offshore renewable energy systems, primarily driven by wind and tidal power, offer a sustainable and scalable solution to the growing global energy demand. However, the inherent variability and intermittent nature of these resources present significant challenges for maintaining grid stability and reliability. Effective generation control (GC) and LFC are critical for addressing these challenges, ensuring a stable supply of electricity while integrating these offshore systems into existing grids. The dynamic marine environment, characterized by fluctuating wind speeds, tidal flows, and environmental conditions such as saltwater corrosion and extreme weather events, necessitates robust control strategies and advanced system designs to achieve reliable operation.

Recent advancements in control methodologies have significantly enhanced the robustness of offshore energy systems. FO controllers, with their superior ability to handle complex and dynamic systems, have emerged as a preferred choice for frequency stabilization in offshore networks. These controllers offer improved flexibility and precision over conventional integer-order designs, enabling better adaptation to the dynamic behavior of offshore renewable sources. Furthermore, intelligent control techniques, such as Artificial Neural Networks (ANN), Fuzzy Logic Controllers (FLC), and hybrid methods, have been employed to optimize the real-time management of power output and frequency regulation. Predictive and adaptive control strategies, leveraging real-time measurements and resource estimation, allow dynamic adjustments to generation and load, ensuring operational stability under varying environmental conditions.

The integration of offshore power systems into mainland grids often relies on High Voltage Direct Current (HVDC) technology, which provides efficient and reliable power transmission over long distances. HVDC systems also enhance frequency regulation by employing advanced converter technologies capable of fast and precise adjustments to power flows. Decentralized LFC strategies, particularly suitable for offshore setups, ensure localized frequency management without over-reliance on centralized control, thereby mitigating the risks associated with communication delays and single points of failure. Additionally, multi-area LFC approaches enable coordinated control of interconnected offshore and onshore systems, ensuring system-wide stability.

ES systems play a pivotal role in complementing LFC efforts in offshore environments. Technologies such as batteries, flywheels, and pumped hydro storage systems provide a buffer to mitigate short-term fluctuations in power generation, improving the overall robustness and reliability of offshore power systems. These storage systems, when coupled with advanced optimization techniques like Grey Wolf Optimization (GWO) and Firefly Algorithms, further enhance the efficiency of control strategies by enabling optimal parameter tuning and resource utilization.

Looking ahead, the integration of emerging technologies such as wave energy converters and hybrid offshore platforms combining wind, tidal, and solar power holds immense potential for diversifying offshore energy portfolios. By incorporating state-of-the-art control algorithms and robust system designs, offshore renewable energy systems can significantly contribute to a resilient, sustainable, and low-carbon energy future. These advancements underscore the importance of continued research and innovation in control methodologies to address the unique challenges of offshore power generation and ensure its seamless integration into global energy networks.

To establish a coherent connection between the different research efforts and to summarize the significant contributions in generation control, the following tables categorize the literature based on key themes, including Automatic Gain Control (AGC) advancements, renewable energy integration, nuclear energy systems, and hybrid power systems. These tables provide a structured overview of the evolution of control strategies, highlighting the progression from traditional methods to modern, sophisticated approaches that address the complexities of contemporary power systems, thereby ensuring a seamless integration of various energy sources and optimizing grid stability and efficiency.

### Recent advances in optimization techniques

This This literature review examines recent advancements in optimization techniques applied to a wide array of engineering fields, including energy systems, manufacturing processes, and neural networks. These studies collectively highlight the substantial benefits of advanced optimization methods in enhancing system efficiency, reliability, and overall performance. Specifically, they underscore how these techniques are crucial in addressing the multifaceted challenges faced by energy grids, particularly in developing regions such as India and Africa, where grid reliability remains a major concern.

In the context of energy systems, Alrasheed et al.^94^ conducted a comparative study on optimizing abrasive water jet machining for glass-carbon fiber reinforced composites using evolutionary optimization techniques. Their work emphasizes how optimization enhances machining performance, a concept similarly applicable to improving efficiency in energy generation and distribution systems. Restrepo-Cuestas et al.^95^ applied metaheuristic optimization techniques for photovoltaic cell parameter estimation, an area critical to enhancing solar energy utilization, especially for countries like India, which are expanding their solar capacity, and for regions in Africa where solar energy presents a viable solution for improving access to electricity.

Abdelhakeem et al.^96^ addressed the operational challenges posed by wind energy uncertainties in power systems, focusing on the unit commitment problem. This is particularly relevant in the Indian context, where integrating large-scale wind power into the grid presents challenges due to its intermittency. Similarly, in Africa, where wind energy potential is abundant, optimizing its integration into national grids is crucial to ensure reliability and stability, thus preventing grid failures and blackouts. Focusing on neural networks, Raiaan et al.^97^ reviewed hyperparameter optimization techniques for Convolutional Neural Networks (CNNs), offering insights that can be adapted to optimize smart grid systems, where machine learning can enhance decision-making processes in energy management and fault detection.

Thirunavukkarasu et al. (2022) expanded on the role of optimization techniques in microgrid energy management systems, emphasizing their significance in balancing supply and demand, reducing costs, and improving system efficiency. Such approaches are particularly pertinent to both India and Africa, where microgrids can provide reliable power to rural and off-grid areas. Yuan et al.^98^ and Alqahtani et al.^99^ explored the application of the osprey optimization algorithm for parameter estimation in renewable energy systems, including PEMFCs and lithium-ion batteries. These approaches are crucial in both regions for improving the efficiency and reliability of energy storage systems that support intermittent renewable energy sources like solar and wind.

In the realm of voltage regulation, Pazhanimuthu et al.^100^ explored the use of zebra optimization algorithms in Automatic Voltage Regulator (AVR) systems, which are vital for stabilizing voltage levels in power systems. This optimization can enhance grid stability in both Indian and African grids, where voltage fluctuations are common, especially during peak demand periods or when renewable generation is low.

The growing adoption of solar-powered systems in regions like India has led to optimization studies in solar energy systems, such as the work by Anshory et al.^101^, which optimized a DC-DC boost converter for Brushless DC (BLDC) motor drives powered by solar panels. Their work highlights the importance of optimization in enhancing the performance of solar-powered devices, a key factor in India’s push toward expanding solar energy usage. Similarly, Yuan et al.^102^ optimized hybrid renewable energy systems using the firefly algorithm, taking into account battery degradation and load uncertainties. This study has particular significance in African countries, where hybrid systems integrating solar and wind with storage are becoming increasingly popular to meet energy demands in remote and underserved areas.

Ahsan et al. (2024)^103^ examined the optimal sizing and cost analysis of hybrid energy storage systems for electric vehicles (EVs), utilizing metaheuristic PSO and firefly algorithms. Their findings offer insights into integrating advanced storage solutions into the transportation sector, which is particularly relevant in countries like India and Africa, where the adoption of electric vehicles is growing as part of efforts to reduce emissions and reliance on fossil fuels.

Further research has explored the integration of optimization techniques in multi-area power systems. A study in^104^ analyzed a 2-area, 4-unit power plant to enhance power transmission resilience during disturbances. The research compared the effectiveness of various control strategies, including 4-FOPID-PSO, PID-PSO, and PI controllers. The 4-FOPID-PSO controller emerged as the most effective in stabilizing frequency and damping oscillations, thereby preventing blackouts and supporting a cleaner energy approach by integrating nuclear power. In countries like India, which are transitioning toward cleaner energy sources, such strategies are crucial for achieving grid stability and minimizing environmental impacts.

Despite these advancements, several gaps remain, particularly in the integration of green energy systems in developing countries. As India and African nations expand their renewable energy capacity, they face unique challenges related to grid stability, the intermittency of renewable sources, and infrastructure limitations. To address these challenges, further research is needed to explore advanced optimization strategies and control mechanisms that can ensure reliable and resilient power systems, while supporting a sustainable transition to green energy.

The integration of advanced optimization techniques into the energy systems of India and Africa will be critical in addressing the unique challenges these regions face. These include the development of energy storage systems, optimization of hybrid power systems, and the implementation of smart grid technologies. As renewable energy penetration increases, the need for optimization frameworks that can balance grid supply and demand, manage intermittent energy sources, and minimize environmental impacts will be paramount. The future of energy systems in both India and Africa depends on these advanced strategies, which can enable sustainable development while ensuring reliable access to electricity for all.

### Research gap

#### Green energy

While the literature extensively explores various aspects of the transition to green energy, including regulatory frameworks, control strategies, and the role of nuclear energy, several key research gaps persist:

- **Integration of Socio-Economic Factors in Regulatory Pathways:** Although Aydin et al.^59^ emphasize the need for holistic regulatory frameworks that incorporate socio-economic factors for sustainable environmental management, there is limited research on how these frameworks can be effectively implemented across diverse socio-political contexts. Further studies are needed to develop adaptable regulatory models that are tailored to specific regional or national characteristics, promoting green energy transitions in varied settings.
- **Advanced Control Strategies for Low-Carbon Systems:** Shi et al.^60^ highlights the efficiency of advanced control strategies in CCHP systems. However, research focusing on the scalability and adaptability of these strategies across different renewable energy systems and varying scales of operation remains underdeveloped. Investigating the performance of advanced control strategies in diverse renewable energy infrastructures, particularly in off-grid or decentralized systems, would be valuable.
- **Role of Nuclear Energy in Renewable Hybrid Systems:** While studies such as Aktekin et al.^74^ assess the techno-economic viability of grid-connected nuclear and renewable hybrid systems, there is a lack of research on the long-term environmental and economic impacts of these hybrid systems. Additionally, the social acceptance and public perception of integrating nuclear energy with renewable sources in different cultural and socio-economic settings remain under-explored.
- **Optimization of Renewable Energy Storage Systems:** The optimization of energy storage systems in renewable energy frameworks, as discussed by Kaan et al.^84^, is critical for enhancing the reliability and sustainability of green energy systems. However, research on the lifecycle costs, environmental impacts, and scalability of various storage technologies, particularly in developing countries or regions with limited infrastructure, is insufficient.
- **Challenges in Green Hydrogen Production and Utilization:** Studies such as those by Awad et al.^89^ and Garcia and Oliva (2023)^105^ highlight the potential of green hydrogen production using renewable sources. However, there is a gap in understanding the economic and logistical challenges associated with large-scale production, storage, and distribution of green hydrogen. Further research is needed to develop cost-effective and efficient strategies for integrating green hydrogen into existing energy grids and industrial applications.
- **Technological Advancements in Wind Energy Systems:** Although Liu et al.^93^ explore the impact of turbine technology on wind energy potential and CO2 emissions, more research is required to evaluate the long-term reliability, maintenance challenges, and environmental impacts of emerging wind turbine technologies. Additionally, the potential for combining wind energy with other renewable sources, such as solar or tidal energy, to create synergistic effects in reducing carbon emissions has not been fully explored.
- **Regional Variations in Renewable Energy Potential:** Hassan et al.^88^ and Abdullah-Al-Mahbub et al.^106^ provide insights into the renewable energy landscape in Europe and Bangladesh, respectively. However, there is a need for more granular studies that assess renewable energy potential, challenges, and opportunities at regional and local levels, especially in under-researched regions with unique environmental and socio-economic conditions.
- **Environmental and Economic Viability of Surplus Renewable Energy Utilization:** De Souza et al.^91^ discuss the economic viability of using surplus energy for green hydrogen production. Yet, comprehensive research on the optimal use of surplus renewable energy across different geographic and economic contexts is lacking. Studies comparing the feasibility of various utilization strategies, such as energy storage, grid integration, and hydrogen production, are necessary to inform policy and investment decisions.

Addressing these research gaps is crucial for advancing the integration of green energy technologies and ensuring a sustainable energy future. The identified areas call for a holistic approach that considers socio-economic, technological, and environmental dimensions to foster effective green energy transitions.

#### Generation control

From the analysis of Tables 1, 2, 3, and 4, the following research gaps have been identified and categorized into three primary areas:

**Table 1:** Key contributions in AGC and generation control.

Reference	Author	Year	Major contributions/synthesis	Future scope
^107^	Dulau, Mircea, and Dorin Bica	2014	Mathematical modelling and simulation of steam turbine behaviour is studied by authors	• Improve model accuracy and real-world applicability
^108^	A. Kachhwaha, S. K. Pandey, A. K. Dubey, and S. Gupta	2016	Multi area system with multi source power (thermal, nuclear, hydro, etc.) with EV loading is controlled through Fractional order controller by authors	• Investigate different type of loadings or disturbances for enhanced generation control. Controller has not been properly tuned. Hence optimization technique ca be utilized for improved control
^109^	A. H. Mazinan and M. F. Kazemi	2010	Fuzzy-based predictive scheme for load–frequency control is studied by authors	• Step loading has been considered in the paper. Real pattern disturbances can be considered
^110^	N. Gupta, N. Kumar and N. Singh	2018	PSO-tuned AGC strategy with SMES in multi-source power system	• Implementation of new meta heuristic techniques can be utilized for improving the response of the system
^111^	M. K. Debnath, T. Jena, and R. K. Mallick	2016	PD-PID cascaded controller optimized by GWO for AGC in multi area thermal system	• Tilt controllers or fractional order controller can be applied for improved control with increased complexity in system
^112^	S. Singh and R. Mitra	2014	2-area system with multi source power is controlled using PID controller tuned to PSO	• New optimization techniques with tilt controllers can be applied with increased complexity
^113^	P. Kumar and A. K. Bohre	2021	Optimization-based planning for solar-PV and STATCOM	• Integration of additional renewable energy sources
^52^	Kumar, P., and A.K. Bohre	2022	Optimal resource allocation using PSO with a multi-objective approach	• Dynamic allocation of resources in real-time grid operation
^114^	J. Gholinezhad, M. R. Safari, and T. R. Noroozian	2011	Controller Design for Load Frequency Stabilization Utilizing magnetic and capacitive energy storage	• Integration of additional energy storage technologies
^115^	P. Panda and A. K. Nanda	2018	Development and Evaluation of a 3-DOF PID Controller for LFC	• Performance of 3-DOF controllers in larger interconnected systems
^116^	H. Li, Y. Li, M. Yang, and D. Li	2022	AGC strategy using fuzzy PID control with parameter optimization	• Application of fuzzy control strategies in different scenarios
^117^	K. Günther and C. Sourkounis	2022	Control strategy for variable speed wind turbines for grid stability	• Integration of renewable energy sources into the grid
^118^	J. An and X. Tang	2022	Event-triggered adaptive control strategy for load frequency control	• Use of event-triggered control in real-time grid operation
^119^	K. Akter, L. Nath, T. A. Tanni, A. S. Surja, and M. S. Iqbal	2022	Improved LFC strategies for single and multi-entity systems	• Scalability of the proposed strategy in larger power systems
^120^	M. S. Redoy and Ruma	2022	Implementation of PSO tuned PID controller for LFC	• Effectiveness of PSO-based controllers in different interconnected systems
^121^	Elsisi, M	2021	Development of a low computational burden Model Predictive Control (MPC)	• Application of the proposed control strategy in autonomous vehicles
^122^	Elsisi M, Tran M-Q, Hasanien HM, Turky RA, Albalawi F, Ghoneim SSM	2021	Development of robust MPC for AVR	• Integration of optimization algorithms in voltage regulation
^123^	M. Elsisi	2020	Development of Varying Structure Control for Power network in Nuclear Reactors network	• Performance of variable structure control in different reactor scenarios
^124^	Elsisi, M	2022	Enhancement of Grey Wolf algorithm with opposition and quasi-learning approaches	• Application of the enhanced optimizer in various optimization problems
^125^	Elsisi, M	2020	Design of a nonlinear model predictive controller using a modified multitracker optimization algorithm	• Application of the controller in different systems
^126^	Elsisi, M	2021	Development of a non-fragile PID controller	• Robustness and applicability of the controller in various scenarios
^57^	Elsisi, Mahmoud et al	2015	Design of a PID controller using the ABC algorithm for load frequency control	• Performance of the controller with varying nonlinearities
^127^	Elsisi, Mahmoud et al	2020	Design of a battery charge management controller for hybrid PV/wind systems	• Integration of advanced battery management techniques
^58^	V. Yarlagadda, G. Lakshminarayana, M. Nagajyothi and I. Neelima	2022	Implementation of Fuzzy control for frequency control and stability improvement	• Effectiveness of Fuzzy control in maintaining grid stability

**Table 2:** Literature survey for generation control in renewable energy integrated power system.

Reference	Author name	Year	Major contributions/synthesis	Future scope
^128^	Diambomba Hyacinthe Tungadio, Yanxia Sun	2019	A detailed review for Load frequency controllers considering renewable energy integration in power system has been presented by authors	• Investigate the impact of different renewable energy sources on load frequency control • Develop advanced control strategies for improved performance in renewable-integrated power systems
^129^	Jizhen Liu, Qi Yao, Yang Hu	2019	MPC for LFC of hybrid power network with wind power and thermal power has been studied by authors	• Explore the integration of additional renewable sources in hybrid power systems • Optimize control algorithms for enhanced load frequency stability and efficiency
^130^	Bheem Sonker, Deepak Kumar, Paulson Samuel	2019	Dual loop internal mode controller for LFC issue in renewable energy integrated hydrothermal system is proposed by authors	• Extend the application of the dual loop IMC structure to address load frequency control in different loading condition. Only step loading has been considered in the proposed paper
^131^	Deepesh Sharma, Naresh Kumar Yadav	2019	LFC with Lion Algorithm with Levy Update is proposed for multi area multi source system	• Enhance the performance and robustness of the control scheme with new optimization technique
^132^	Gulzar, M.M	2023	Hybridized MPC-(1 + PIDN) controller for solar, wind and hydro thermal system is proposed	• New renewables can be added for increased complexity in the system and (1 + PIDN) controller can be replaced by fractional order controller for improved performance of the system
^133^	Iqbal, M., & Gulzar, M. M	2022	A master–slave control design for frequency regulation in multi power network operating under complex conditions	• Expand research to address grid integration challenges in various hybrid power systems, including microgrids and distributed generation.—Investigate adaptability in different scenarios and varying environmental conditions
^134^	Gulzar, M. M., Sibtain, D., Ahmad, A., Javed, I., Murawwat, S., Rasool, I., & Hayat, A	2022	Efficacious design of adaptive MPC for LFC in multi power network	• Develop methods for real-time adaptation and reconfiguration of control parameters based on system dynamics.—Investigate the integration of AI and machine learning techniques for intelligent load frequency control in hybrid power systems
^135^	Latif, A., Suhail Hussain, S. M., Das, D. C., & Ustun, T. S	2021	Optimization of a dual-stage controller for stabilized frequency in a maritime microgrid system powered by hybrid wind and ocean wave energy sources	• Explore the feasibility and effectiveness of double stage controller optimization in various maritime microgrid scenarios.—Investigate the potential integration of additional renewable energy sources, such as ocean wave energy, into the system.—Optimize control strategies for improved stability and performance in maritime microgrid systems

**Table 3:** Studies on nuclear energy enriched systems.

Reference	Author name	Year	Major contributions/synthesis	Future scope
^136^	N. Muppoori, N. Dasari and Y. G	2021	The study compares PID and disturbance rejection based control (DRBC) for AGC in a multi-source power network, showing DRBC’s superior adaptability and stability under dynamic conditions has been studied by authors	• Investigate real-world applicability of ADRC in power systems and explore adaptive control methods for improved performance
^137^	M. K. Bhaskar, N. S. Pal and V. K. Yadav	2018	A thorough analysis of AGC with PID, Fuzzy, and neuro fuzzy based Control in Multi-entity power network has been studied by authors	• Enhance control techniques for multi-area power systems and investigate hybrid control strategies for improved system stability
^138^	V. Pavlovsky, A. Steliuk, O. Lenga, V. Zaychenko and M. Vyshnevskyi	2017	Modelling of LFC incorporating under frequency load shedding relays, protection systems, and AGC models has been studied by authors	• Extend the model’s practicality by including a broader range of protection systems and hardware-in-the-loop (HIL) simulations. Apply the simulation in real power systems and validate the results in field conditions
^139^	Dhanasekaran, Boopathi, Saravanan Siddhan, and Jagatheesan Kaliannan	2020	Utilization of ant colony optimization for LFC in a single entity nuclear power network	• Assess the adaptability and scalability of ant colony optimization to more complex multi-area power systems and investigate real-time implementation
^140^	Boopathi, D., Jagatheesan, K., Samanta, S., Anand, B. and Satheeshkumar, R	2023	Integration of Energy Storage (ES) Units for LFC in Nuclear Generators-Based energy network	• Optimize the sizing and placement of energy storage units and explore advanced energy management strategies. Investigate grid-connected storage solutions
^141^	Murugesan, D., Jagatheesan, K., Kulkarni, A.J., Shah, P	2023	Development of a socio-inspired technique for LFC in nuclear power plants using Cohort Intelligence algorithm-Based PID Controller	• Critically evaluate the robustness and real-world applicability of socio-inspired techniques. Integrate with advanced optimization methods and adapt to different power plant configurations
^142^	Paliwal, N., Srivastava, L., Pandit, M., Singh, P	2022	Implementation of Jaya Algorithm-Based LFC incorporating ES in Nuclear Power Plants	• Assess the scalability of the Jaya algorithm in larger power systems and its performance under varying grid conditions. Investigate the optimal sizing and placement of energy storage systems and extend robustness and adaptability assessments

**Table 4:** Literature survey for generation control in wind power enriched hybrid power system.

Reference	Author name	Year	Major contributions/synthesis	Future scope
^143^	Pati, Subhranshu Sekhar, Sambit Dash, and Saroj Kumar Mishra	2018	Authors introduced Fuzzy tuned PI controller for LFC in Multi Entity System with Wind Energy	• Investigate the performance under varying wind conditions with advanced control strategies
^144^	Zhangs, Shiyu, Zongxiang Lu, and Ying Qiao	2021	Authors developed Data-Driven Technology for Frequency Regulation Parameter Identification in Wind Farm AGC	• Advance data-driven techniques for accurate parameter identification in diverse wind farms and larger systems
^145^	Oshnoei, Arman, et al	2018	A detailed review of the different control strategies in Wind Farms for LFC is done. Different level of wind penetration is injected and corresponding robustness of the controller has been analyzed with fractional order controller by authors	• Explore advanced control strategies and consider hybrid systems with wind energy for diverse climatic conditions
^146^	Aziz, Asma, Aman Than Oo, and Alex Stojcevski	2018	Authors analyzed the Impact of Frequency-Sensitive Wind Plant Penetration on LFC in Hybrid Power Systems. Simulation shows that 60% wind power penetration is possible with low load disturbance	• Intelligent control strategies are required for handling higher order disturbances with frequency sensitive wind power plant
^147^	Kheshti, Mostafa, et al	2021	Improved Frequency Regulation in Wind-Integrated Multi-Area Systems using Lightning flash algorithm-Fuzzy PID Control	• Identification of critical parameters in wind power system such as pitch angle for optimization for improved control
^148^	Begum, Benazeer, et al	2020	Authors designed and implemented Fuzzy Logic tuned PID Controllers for LFC in Power Systems Incorporated with Wind Farms	• Evaluate the effectiveness of alternative control strategies for wind-integrated power systems and their adaptability to changing wind farm configurations
^149^	Ullah, Kaleem, et al	2022	Investigated Automatic Generation Control in Modern Power Systems with Wind Power and Electric Vehicles	• Research the integration of electric vehicles to enhance grid stability in power systems with wind energy integration and study the interaction of electric vehicles with AGC
^150^	Ramesh, Maloth, Anil Kumar Yadav, and Pawan Kumar Pathak	2021	Authors Conducted an Extensive Review of Load Frequency Control in Solar-Wind-Based Hybrid Renewable Energy Systems	• Investigate the impact of climate change and varying weather conditions on Load Frequency Control in hybrid systems integrating solar and wind sources

Category 1: Power System Disturbances and Optimization Techniques

- **Diverse Disturbances in Realistic Power Systems**: Many studies focus on step load disturbances, which simplify real-world scenarios. In practice, power systems encounter a wide range of disturbances, including variable load demands, environmental changes, and unpredictable outages. There is a need for research on control strategies that effectively manage and mitigate these diverse disturbances in real-time.
- **Need for Advanced Optimization Techniques:** Current optimization techniques, while effective in certain scenarios, may not adequately address the rapid and intelligent damping of fluctuations in modern power systems. There is a pressing need for advanced optimization methods that can provide faster, more adaptive responses to ensure stability and efficiency in power generation and distribution systems.
- **Isolated Nuclear Power System Control:** Few studies focus on the generation control of isolated nuclear power systems, critical for remote or autonomous operations. Research is required to develop control strategies specifically for these isolated nuclear systems, addressing their unique operational challenges to ensure reliable and safe power generation.

Category 2: Hybrid power systems and real-time control

- **Real-Time Complexities in Hybrid Power Systems:** Many hybrid power systems are tested under fixed loading conditions, which do not reflect the complexities of real-world operations. There is a significant gap in research addressing real-time operational challenges, such as fluctuating power demands, integration of multiple energy sources, and dynamic load balancing.
- **Nuclear Power Integration in Hybrid Systems:** The integration of nuclear power into hybrid systems for generation control is underexplored. Research is needed to develop intelligent control strategies that manage real-time disturbances in nuclear-enriched hybrid systems, ensuring stable and efficient operation under varying conditions.
- **Wind Power Integration and Control:** Wind power is inherently uncertain due to its dependence on climatic conditions. Although there has been progress in integrating wind power into multi-area hybrid systems, there is a lack of intelligent control strategies that enhance the management and utilization of wind power. Further research should focus on developing these strategies to improve the reliability and efficiency of wind-integrated systems.

Category 3: Wind energy utilization and control

- **Resilience of Modern Power Systems to Wind Energy Variability:** Wind energy, while abundant, is highly variable. Modern power systems must be resilient enough to efficiently extract and transmit wind energy under different weather conditions. Robust systems and control strategies that can adapt to these variations are needed to ensure a consistent energy supply from wind sources.
- **Optimization of Wind Energy Extraction through Pitch Angle Control:** The pitch angle of wind turbines plays a crucial role in optimizing wind energy extraction. However, its potential in generation control within wind-integrated multi-area hybrid systems is underexplored. Research is needed to optimize pitch angle control mechanisms, enhancing the efficiency and reliability of wind power generation.

Addressing these research gaps in generation control will enhance the resilience and efficiency of modern power systems. By developing advanced and adaptive control strategies, the integration of diverse energy sources can be optimized, paving the way for more robust and sustainable power systems.

#### Optimization techniques

Despite the significant advancements in optimization techniques across various engineering applications, several research gaps remain:

- **Scalability and Generalization of Optimization Techniques:** While studies like those by Alrasheed et al.^94^ and Restrepo-Cuestas et al.^95^ show the effectiveness of specific optimization techniques in certain applications, research on scaling these techniques to other complex systems and larger-scale industrial applications is limited. Further studies are needed to explore how these methods can be adapted for broader applications with higher dimensionality or more intricate constraints.
- **Integration of Optimization Techniques with Real-Time Systems:** Abdelhakeem et al.^96^ and Thirunavukkarasu et al. (2022)^151^ highlight the role of optimization in energy management and power systems. However, there is limited exploration of how these techniques can be integrated into real-time systems for dynamic and adaptive control. Developing optimization algorithms that operate in real-time, handling rapidly changing conditions and uncertainties, is a crucial area for future research.
- **Optimization Under Uncertainty:** While some studies focus on parameter estimation and uncertainty in fuel cells and battery models, there is a gap in addressing optimization under uncertainty across other critical domains. Future research should explore robust optimization techniques that account for uncertainties in data, modelling, and external conditions, particularly in renewable energy systems and autonomous technologies.
- **Hybrid Optimization Approaches:** Although metaheuristic and hyperparameter optimization techniques are discussed in the context of energy storage systems and neural networks, there is a need for more research into hybrid optimization approaches that combine different methods, such as merging metaheuristics with machine learning or deterministic methods. These hybrid approaches could provide more comprehensive solutions for complex, multi-objective optimization problems.
- **Economic and Environmental Trade-offs in Optimization:** While some studies consider cost analysis and battery degradation, there is insufficient research on the broader economic and environmental trade-offs in optimizing engineering systems. Future research should focus on frameworks that address these trade-offs, helping decision-makers balance economic feasibility, environmental sustainability, and technical performance.
- **Application of Novel Optimization Algorithms in Emerging Technologies:** Current research often applies optimization techniques to established technologies. There is a lack of exploration of novel algorithms in emerging technologies such as quantum computing, autonomous systems, and advanced manufacturing. Research should investigate the potential of these optimization techniques to enhance the performance and reliability of cutting-edge technologies.
- **Interdisciplinary Optimization Approaches:** The literature tends to focus on domain-specific applications of optimization techniques. There is a gap in interdisciplinary approaches that leverage optimization across different fields, such as combining insights from energy systems, neural networks, and manufacturing processes. Interdisciplinary research could unlock new possibilities for optimization in complex, interconnected systems.
- **Sustainability and Long-Term Impact of Optimized Solutions:** While technical performance is often emphasized, there is a need for more research on the sustainability and long-term impact of optimized solutions. Assessing environmental footprints, lifecycle costs, and societal impacts of optimized systems is essential to ensure they contribute to sustainable development goals.

The future scope for enhancing generation control in homogeneous and hybrid power systems presents numerous opportunities for exploration and innovation. Key areas for future research include:

- **Advancement of Teaching Learning Based Optimization (TLBO) Techniques:** One promising direction is the enhancement of TLBO techniques to handle diverse loading conditions in multi-area systems. Future research should focus on developing hybrid algorithms that integrate TLBO with other optimization methods, addressing complex scenarios with an emphasis on scalability and adaptability for real-time operational changes. To ensure practical implementation, robust simulation models that accurately replicate the dynamic behaviours of large-scale power systems under varied loads are essential.
- **Resilient Control Strategies for Nuclear Energy-Based Power Systems:** For nuclear energy-based power systems, there is a critical need to develop control strategies that meet the high reliability and safety standards required in these contexts. Future research should include emission analysis and environmental impact assessments, integrating these findings into generation control strategies. Simulations under various scenarios, including grid disturbances, can help validate these strategies. Additionally, exploring the integration of renewable energy sources with nuclear power could pave the way for more sustainable hybrid systems.
- **Efficient Control of Multi-Area Hybrid Power Systems:** Managing multi-area hybrid power systems under real-time complexities requires advanced control algorithms capable of adapting to fluctuating power demands and integration challenges, especially in systems involving nuclear power. The incorporation of cyber-physical systems (CPS), artificial intelligence (AI), and machine learning techniques offers promising avenues for improving monitoring, control, and resilience. Furthermore, developing market-responsive control strategies that align with dynamic pricing, demand response, and energy trading within hybrid networks is crucial. Future research should also consider optimization models that balance economic efficiency with environmental sustainability, and explore the integration of distributed energy resources to enhance system flexibility.
- **Robust Control Techniques for Wind Energy-Enriched Hybrid Systems:** In wind energy-enriched hybrid systems, developing robust control techniques using soft computing algorithms is essential for managing random load patterns. Future research should explore hybrid approaches that combine multiple algorithms to create more resilient control systems. Leveraging real-time data analytics and machine learning can further enhance the adaptability and accuracy of these algorithms, while robust testing environments and simulation platforms will be necessary to validate their performance in real-world scenarios.
- **Optimization of Generation Control in Wind-Enriched Systems Under Varied Climatic Conditions:** Future research should focus on utilizing detailed climatic data to refine soft computing techniques for wind power generation. Developing adaptive control systems that respond to changing weather patterns in real-time is vital, along with analyzing long-term climatic trends to enhance system resilience. The optimization of pitch angle control mechanisms is another crucial area, with research needed to maximize energy capture and efficiency. Simulations of extreme weather events can provide valuable insights into the performance and resilience of these control strategies. Additionally, exploring the integration of wind power with other renewable sources can further bolster system stability.
- **Estimation of Optimal Pitch Angles for Wind Turbines in Multi-Area Systems:** Estimating optimal pitch angles for wind turbines is a promising area for future research. Developing data-driven models that incorporate historical and real-time data, coupled with machine learning techniques, can significantly enhance control and efficiency. Field testing and validation of these strategies across diverse environmental conditions will ensure their practical applicability and effectiveness in improving generation control.

By addressing these areas, future research can drive significant advancements in the efficiency, reliability, and sustainability of power systems. This comprehensive approach will not only enhance the performance of current energy infrastructures but also support the development of next-generation power systems that are resilient, adaptive, and environmentally sustainable.

## Solution strategies

To effectively manage and optimize hybrid power systems, this section explores various solution strategies that integrate advanced control mechanisms and optimization techniques. It begins by outlining the structural configuration of hybrid grids, emphasizing the interplay of diverse energy sources and the importance of robust control strategies. Advanced optimization methods such as TLBO and PSO are highlighted for their ability to enhance performance and system stability. The section further details the design of dynamic control mechanisms that adapt to the complex demands of hybrid systems, ensuring efficient energy distribution and reliable operation. A comprehensive single-line diagram of the test system is also provided, showcasing critical elements like electromotive forces (EMFs) and line impedance, which are pivotal in managing power flow and maintaining balance within the grid. These strategies collectively aim to address the challenges of hybrid power systems, fostering a sustainable and resilient energy infrastructure.

**Fig. 3:**
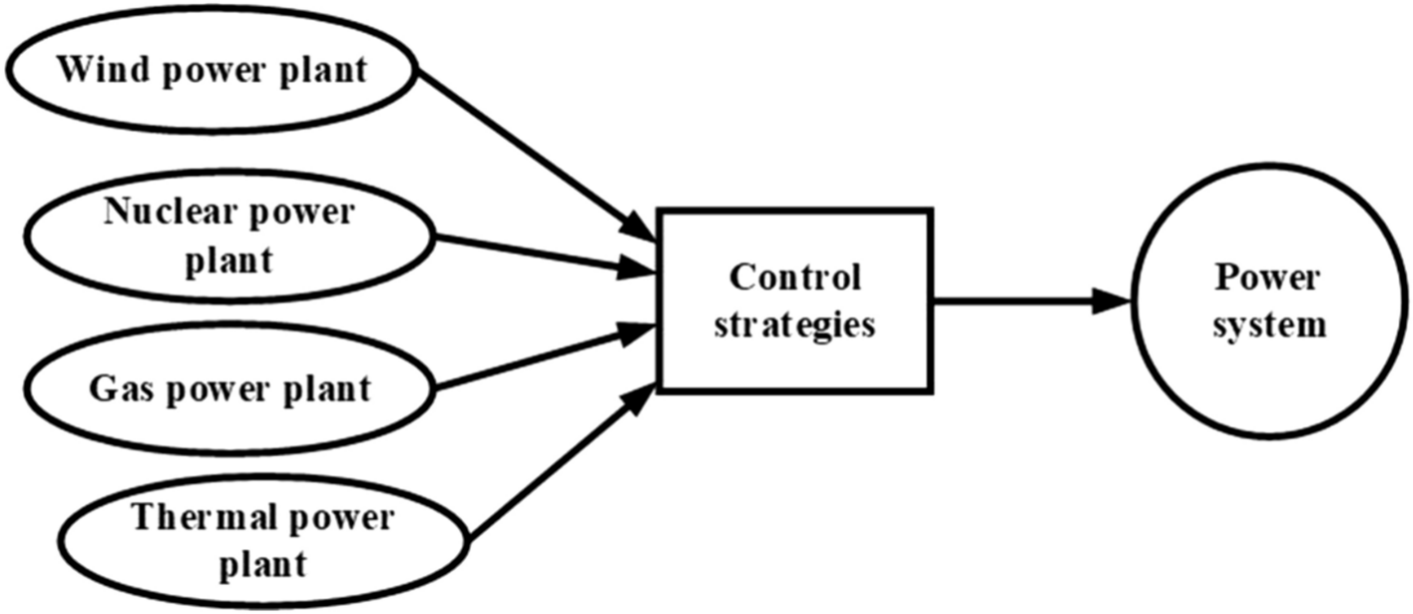
Overview of system considered.

The overall structure of the hybrid grid, along with the power system and control strategies, is depicted in Fig. 3. This figure illustrates how various power plants, including wind, nuclear, gas, and thermal power plants, are integrated into the power system through a centralized controller. The controller coordinates the output from these diverse energy sources, managing their operation to optimize the performance, stability, and efficiency of the entire power system. This integrated approach ensures that the hybrid grid can effectively balance the generation from multiple sources, catering to dynamic energy demands and maintaining grid stability.

**Fig. 4:**
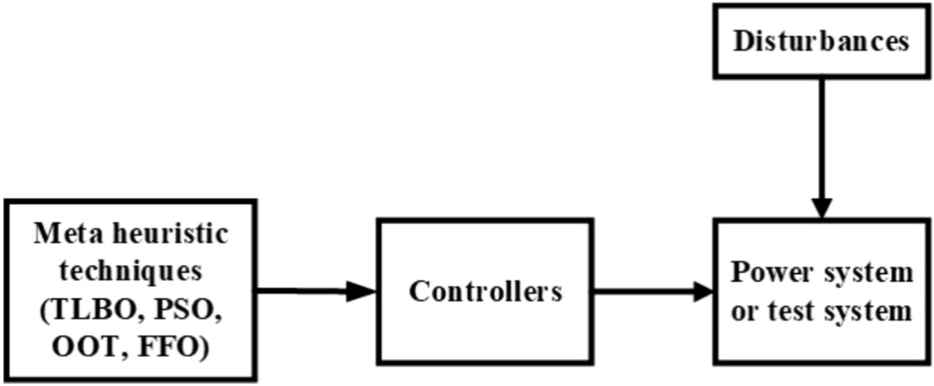
Control Strategies.

Figure 4 illustrates the control strategies employed in power systems, emphasizing the use of metaheuristic techniques such as TLBO, PSO, Osprey Optimization Technique (OOT), and FFO. These optimization methods are utilized to enhance the effectiveness of controllers in managing power systems or test systems. The diagram shows that disturbances can impact the power system, and the optimized controllers, powered by these advanced techniques, play a critical role in mitigating the effects of these disturbances. This setup underscores the importance of leveraging adaptive and robust control strategies to ensure the stability and reliability of power systems under varying conditions.

**Fig. 5:**
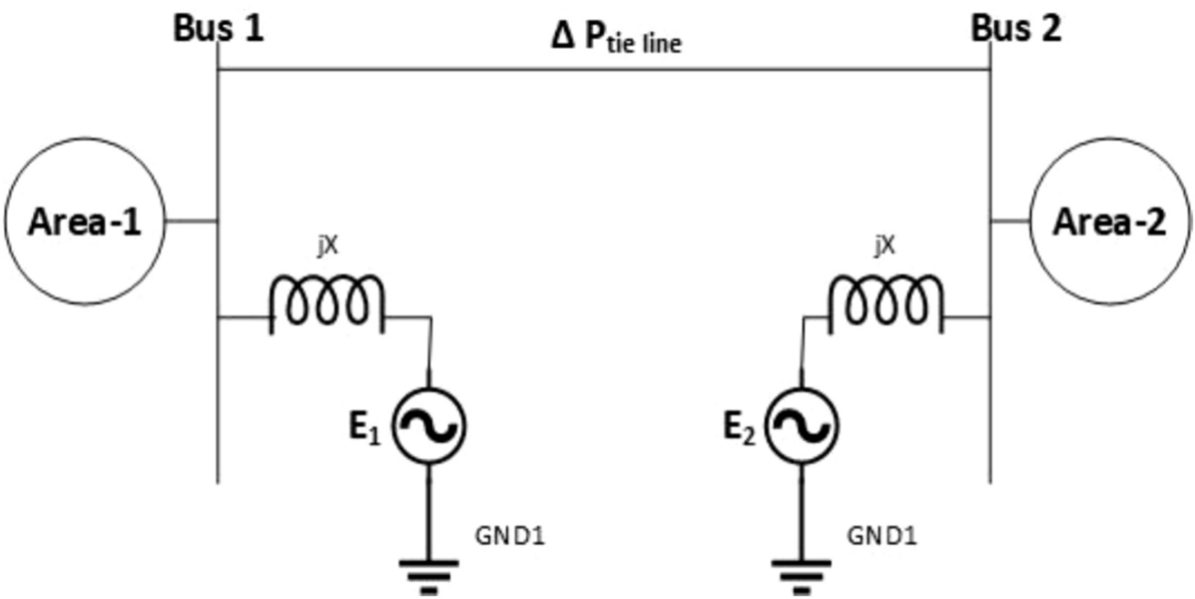
Single line diagram of 2-area network.

Figure 5 illustrates the single-line diagram of a 2-area network consisting of two interconnected regions, Area-1 and Area-2, each linked to its respective power bus (Bus 1 and Bus 2). Each area houses a generator, modelled as voltage sources E_1_ and E_2_, with both grounded at GND1. The system includes inductive reactance, denoted as jX, between the generators and their respective buses, which represents the line impedance and plays a critical role in determining the power flow dynamics within the system.

The power exchange between the two areas occurs through the tie line, represented by the line connecting Bus 1 and Bus 2. The power flow across the tie line is denoted as $\Delta {P}_{tieline}$, which indicates the dynamic power transfer between Area-1 and Area-2. This tie line power exchange is crucial for maintaining the balance and stability of the interconnected power system, especially under varying load conditions or disturbances. The diagram provides a simplified representation of the test system’s structure, focusing on the essential elements that affect power flow and control between two interconnected regions in a grid.

Tie line power flow equation for 2-area system will be given by Eq. (1):

1 $$\Delta P_{TL} = P_{S} \left( {W_{1} - W_{2} } \right)$$

Where, $\Delta {P}_{TL}$ is change in tie line power flow; P_s_ is synchronizing power coefficient; W_1_ is change in frequency of area-1; W_2_ is change in frequency of area-2.

When a load is added to one of the areas in the system, it induces fluctuations in various parameters, such as frequency and tie line power flow, which affect the performance of the 2-area system. To evaluate and enhance the system’s performance, these fluctuations are represented by a cost or fitness function, which is then minimized using an appropriate control loop and controller. These controllers are hybridized with popular heuristic techniques, such as TLBO and PSO. The fitness function used in this study is the Integral of Time Multiplied Weighted Absolute Error (ITWAE), which effectively captures the deviations in system parameters over time. This function, as described in Eq. (2)^152^, serves as the basis for optimizing the control strategies to achieve improved stability and performance of the 2-area system.

2 $$ITWAE = h_{1} \mathop \smallint \limits_{0}^{t} \left| {\Delta f_{1} } \right|dt + h_{2} \mathop \smallint \limits_{0}^{t} \left| {\Delta f_{2} } \right|dt + \mathop \smallint \limits_{0}^{t} \left| {\Delta P_{TL} } \right|dt$$

h_1_, h_2_ is priority based weighting factor.

For single area loading, h_1_ = h_2_ = 1.

For 2-area loading, h_1_ and h_2_ is given by Eq. (3), Eq. (4), Eq. (5):

3 $$h_{1} = \frac{{\left| {P_{al1} } \right|}}{{P_{tal} }}$$

4 $$h_{2} = \frac{{\left| {P_{al2} } \right|}}{{P_{tal} }}$$

5 $$P_{tal} = { }\left| {P_{al1} } \right|\, + \left| {P_{al2} } \right|$$

where, $\left|\Delta {f}_{1}\right|$ is absolute frequency change in area 1; $\left|\Delta {f}_{2}\right|$ is absolute frequency change in area 2; $\left|\Delta {P}_{TL}\right|$ is absolute tie line power flow change; ${h}_{1}$ is weighted value of area 1; ${h}_{2}$ is weighted value of area 2; ${P}_{al1}$ is added load in area 1; ${P}_{al2}$ is added load in area 2 and ${P}_{tal}$ is total added load in both area.

A brief flow chart for the implementation of proposed methodology is given in Fig. 4.

**Fig. 6:**
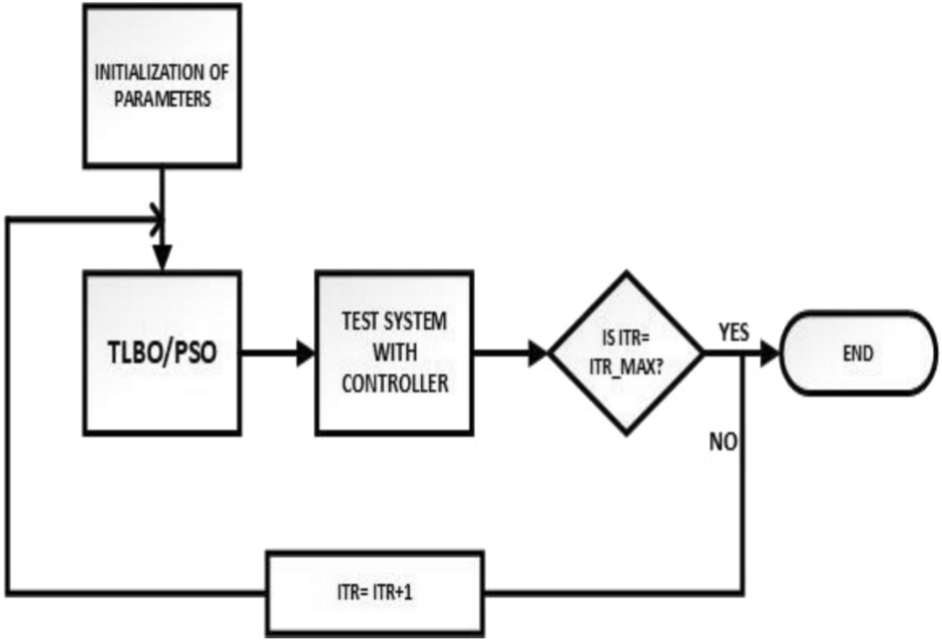
Overview of work flow^152^.

Figure 6 outlines the flowchart of the proposed work, which involves optimizing the performance of a test system using heuristic techniques such as TLBO or PSO. The process begins with the initialization of parameters, which sets the initial conditions for the optimization algorithms. Once parameters are initialized, the TLBO or PSO algorithm is applied to the test system equipped with a controller. The system’s performance is evaluated, and adjustments are made to improve the control strategy. The next step involves checking whether the current iteration (ITR) has reached the maximum number of iterations (ITR_MAX). If the iteration count has not yet reached the maximum, the process increments the iteration count (ITR = ITR + 1) and loops back to apply the TLBO or PSO optimization again.

This iterative process continues until the iteration count equals the predefined maximum number of iterations, at which point the process ends. This loop allows for continuous refinement of the control strategy, leveraging the heuristic optimization methods to minimize the fitness function and enhance the overall performance of the power system under test.

**Fig. 7:**
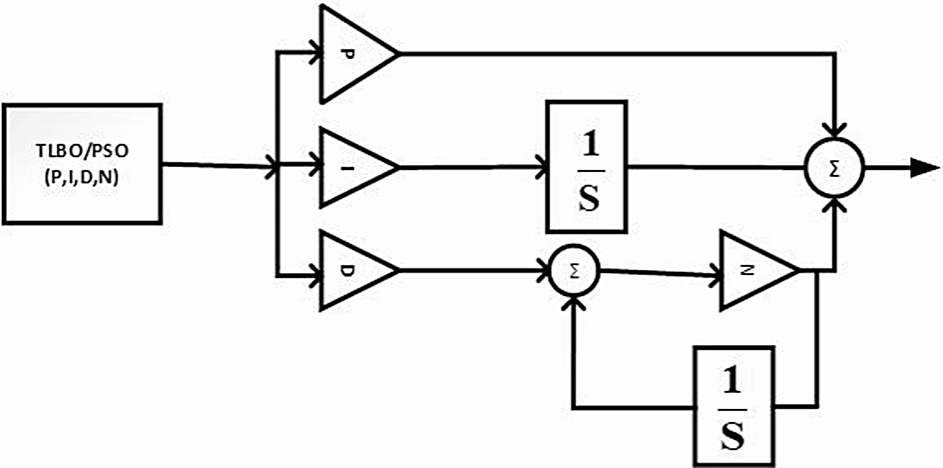
Mathematical framework for tuning controller^152^.

PID controller used in proposed methodology is given in Fig. 7. PID parameters will be obtained from minimizing the designed cost function ITWAE using suitable soft computing technique. its mathematical equation is given as Eq. (6)^152^:

6 $$PID\left( S \right) = P + I \frac{1}{S} + D \frac{N}{{1 + N \frac{1}{S}}}$$

Stepwise execution of TLBO is as follows^152^:

## Teacher phase

a. Initialization of parameters i.e. number of subjects, number of learners.

b. Evaluating mean of result in each subject.

c. Determination of fitness value and identify the best solution.

d. Update the solution based on Eq. (7)

7 $$T_{new} = T_{old} + rand*\left( {T_{teacher} - TF_{mean} } \right)$$

where, $T{F}_{mean}$ is teaching factor.

e. If updated solution is better than old solution then consider it otherwise do not consider.

## Learner phase

f. Now select any two learners i.e. random solutions i.e. ${T}_{i} \ and\ {T}_{j}$.

g. Now substitute these random solutions in fitness function and evaluate it.

h. If ${T}_{i}$ is better than ${T}_{j}$, update learner with Eq. (8)

8 $$T_{new} = T_{old} + rand*\left( {T_{i} - T_{j} } \right)$$

i. If ${T}_{j}$ is better than ${T}_{i}$, update learner with Eq. (9)

9 $$T_{new} = T_{old} + rand*\left( {T_{j} - T_{i} } \right)$$

j. Now, evaluate the fitness function using the updated solution. If it outperforms the existing one, accept it; otherwise, discard it.

k. Continue the process until the maximum number of iterations is reached or the convergence criterion is met.

Figure 5 illustrates the PID controller model strategy enhanced by TLBO or PSO techniques, used in the proposed work. This model integrates Proportional (P), Integral (I), and Derivative (D) control actions, which are fine-tuned using the TLBO or PSO algorithms to achieve optimal performance. In this diagram, the TLBO/PSO block determines the optimal values for the PID parameters (P, I, D) to minimize system errors and improve control efficiency. The optimized parameters are then applied to the respective P, I, and D blocks, which collectively regulate the control signal. The summation points (Σ) represent the combination of the proportional, integral, and derivative outputs, which adjust the control effort to minimize errors over time. The integration blocks labelled as $\frac{1}{S}$ indicate the accumulation of control actions over time, corresponding to the integral function in the PID controller. Additionally, the signal paths show the continuous feedback mechanism where the control actions are adjusted based on real-time system performance. The overall strategy aims to minimize fluctuations and maintain desired system performance by dynamically adjusting the control parameters through iterative optimization with TLBO or PSO. This approach enhances the adaptability and robustness of the control system, ensuring optimal operation under varying conditions and disturbances.

Another fitness function ITMWAE (Integral of Time Multiplied Magnified Weighted Absolute Error) is given in Eq. (10). ITMWAE is handful in increasing convergence rate as per results obtained in^104,153,154^ and^155^.

10 $${\text{ITMWAE}}\, = \,s_{1} \mathop \smallint \limits_{0}^{t} \left| {\Delta f_{1} } \right|dt + s_{2} \mathop \smallint \limits_{0}^{t} \left| {\Delta f_{2} } \right|dt + { }s_{3} \mathop \smallint \limits_{0}^{t} \left| {\Delta P_{tie} } \right|dt$$

11 $$s_{1} = w_{1} \times M;$$

12 $$s_{2} = w_{2} \times M;$$

13 $$s_{3} = w_{3} \times M;$$

Here, M = 10.

Where, M represents the magnification factor; w_1_ = 0.2, w_2_ = 0.5_,_ w_3_ = 0.3; w_1_,w_2_ and w_3_ are weighted values assigned according to priority for frequency and tie-line power deviation; s_1_, s_2_, and s_3_ are magnified weighting factors based on priority, with values being s_1_ = 2, s_2_ = 5, s_3_ = 3 calculated using Eq. (11), Eq. (12) and Eq. (13); $\left|\Delta {f}_{1}\right|, \left|\Delta {f}_{2}\right|$ is absolute frequency deviation; $\left|\Delta {P}_{tie}\right|$ is absolute tie line power deviation; s_1 _and s_2_ are magnified weighted value attached to frequency deviation in area-1 and area-2; s_3_ represents magnified weighted value assigned to the tie line power deviation.

**Fig. 8:**
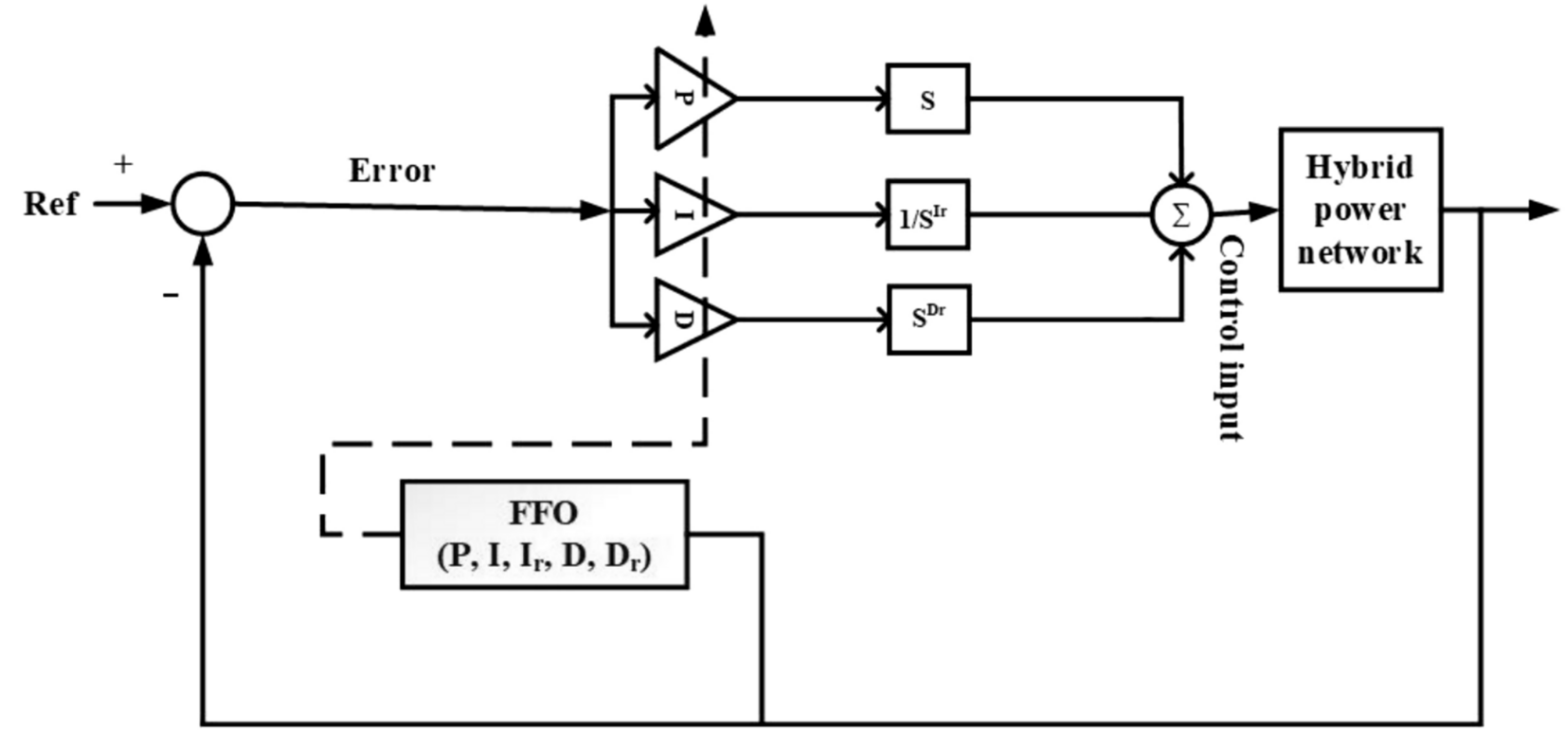
Implementation of fractional order controller.

This fitness function in combination with advance optimization technique such as osprey algorithm^156^, firefly algorithm^155^ can tackle complex problem with complex dynamics. Mathematical execution of fractional order PID (FOPID) controller is given in Fig. 8.

**Fig. 9:**
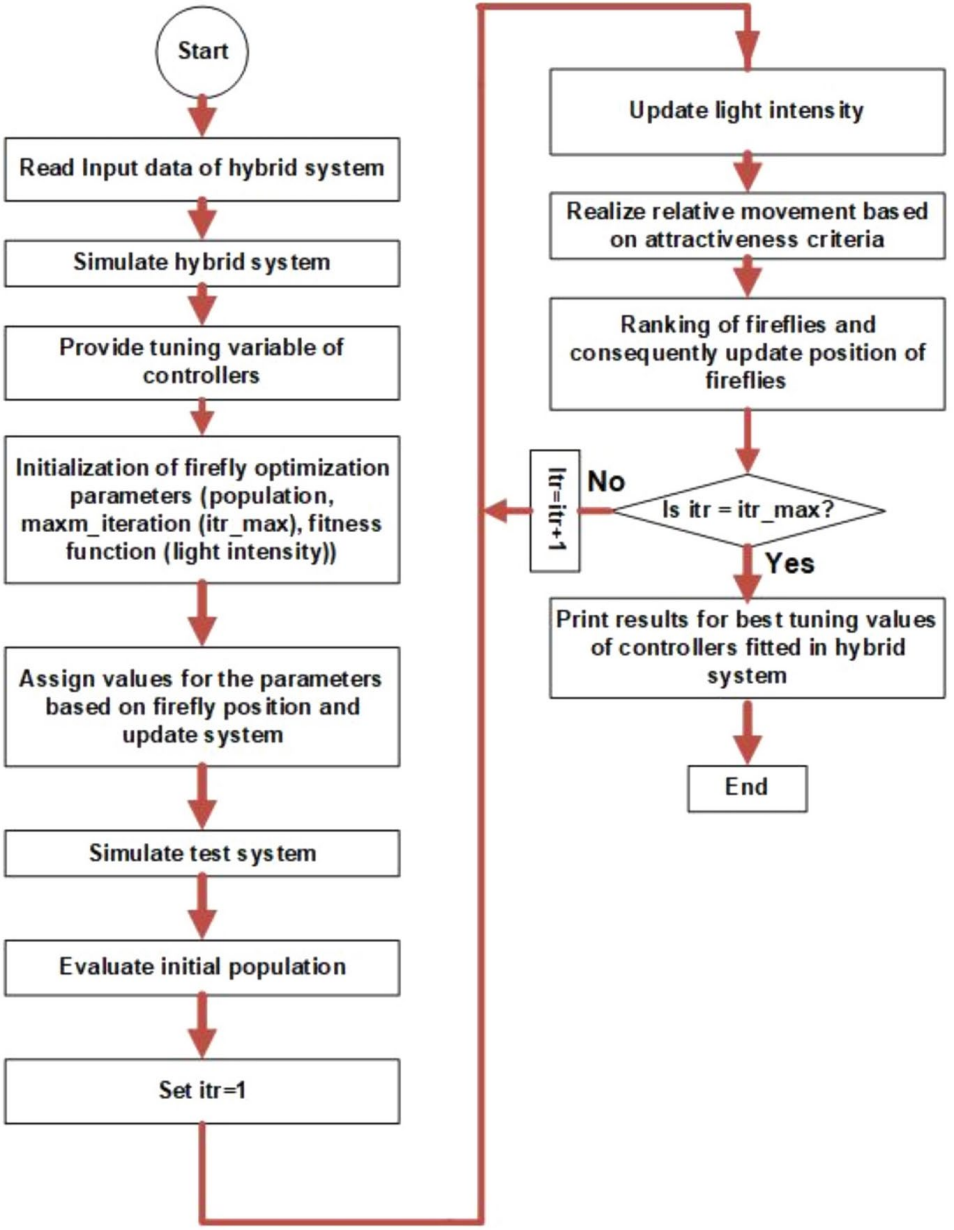
Flowchart for the implementation of firefly algorithm in hybrid network.

Figure 9 presents a flowchart for implementation of firefly algorithm in hybrid power network incorporating wind farm.

### Results considering real time complexities

Case-a: Hybrid test system considering FOPID-FFO controllers (proposed).

Case-b: Hybrid test system considering PID-PSO controllers^157^.

Area-1: Area-1 contains 2-unit power sources namely thermal power plant and gas power plant.

Area-2: Area-2 contains 2-units power sources namely wind power plant and gas power plant.

Case 1: Random loading pattern to be subjected on hybrid power network to test the resilience of proposed technique.

Case 2: Random weather condition is considered to test the resilience of proposed technique.

To validate the effectiveness of the proposed control methodology in the Indian and African energy markets, real-world case studies have been conducted, comparing the performance of fractional-order proportional-integral-derivative (FOPID) controllers optimized using FFO algorithm against traditional PID controllers optimized with the PSO technique. These regions face unique challenges due to rapid renewable energy expansion, fluctuating grid demand, and infrastructure constraints, making advanced control strategies essential for ensuring power system stability. The study is based on a hybrid power system model consisting of two interconnected regions: Area-1, which includes a thermal power plant and a gas power plant, representing the dominant energy sources in India and Africa, and Area-2, which integrates a wind power plant and a gas power plant, reflecting the increasing penetration of renewable energy in these markets.

To test the resilience of the proposed control strategy, two realistic operational scenarios were considered. In Case 1, a random loading pattern was applied to the hybrid power network to assess the system’s ability to maintain frequency stability under fluctuating electricity demand, a common challenge in rural and urban grids in India and Africa due to rapid industrialization and unreliable grid infrastructure. In Case 2, the system was subjected to random weather conditions to analyze the impact of renewable energy variations on power system performance. This is particularly relevant in regions where wind and solar generation are affected by monsoon cycles (India) and seasonal desert winds (Africa).

**Fig. 10:**
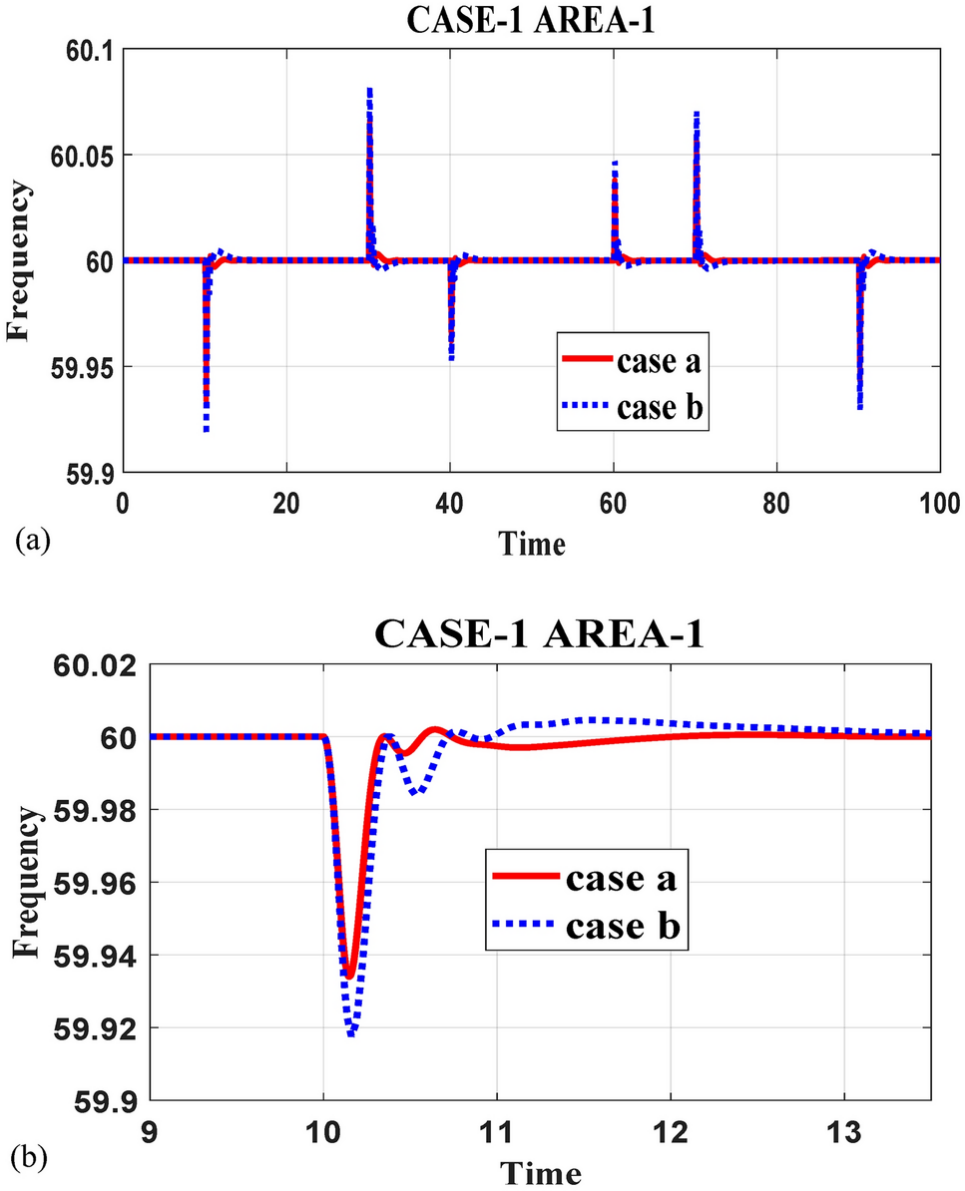
(**a**) Response of frequency over time in area-1 during case-1 (**b**) Zoomed in Response of frequency over time in area-1 during case-1.

**Fig. 11:**
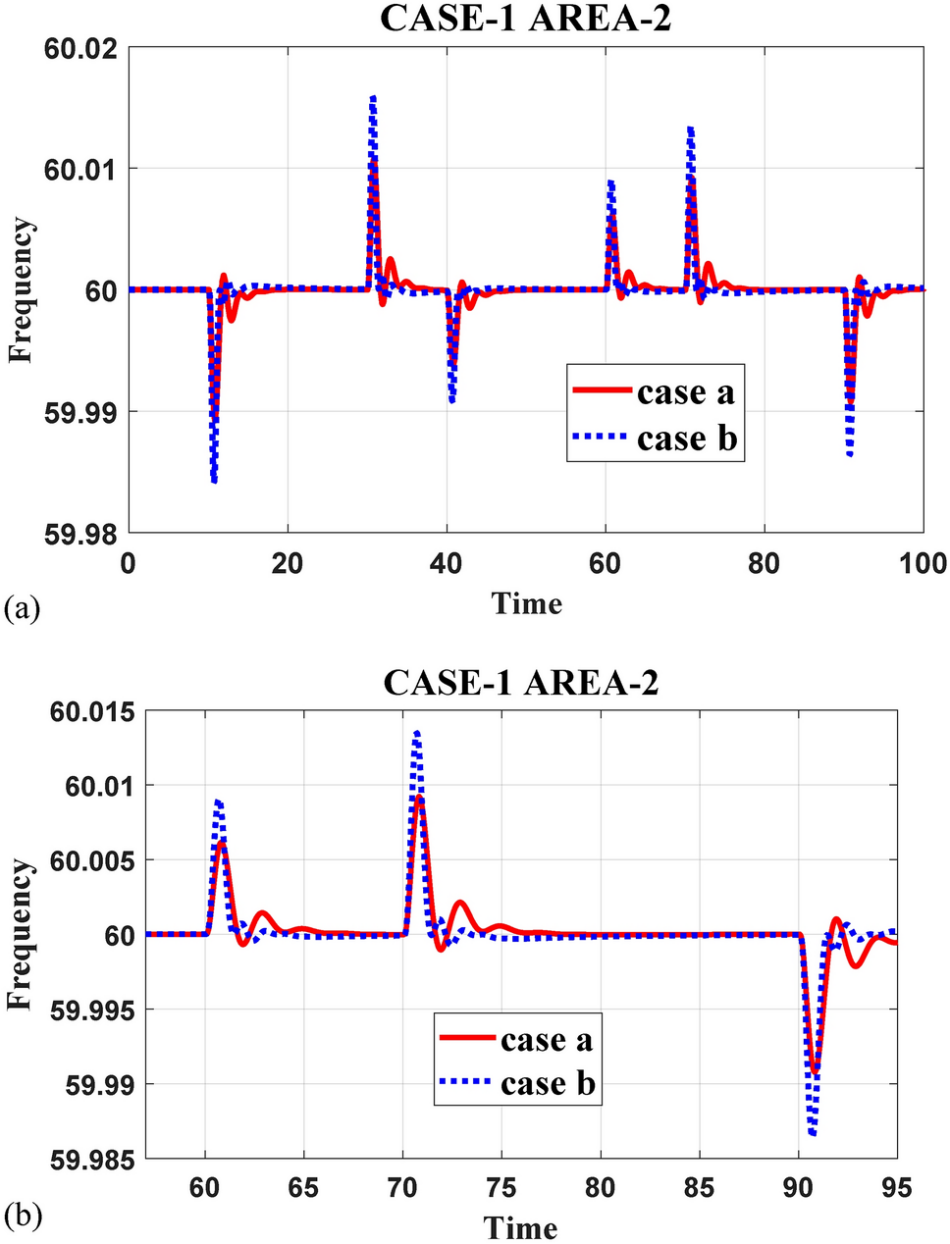
(**a**) Response of frequency over time in area-2 during case-1 (**b**) Zoomed in Response of frequency over time in area-2 during case-1.

The frequency response results for Case 1, as depicted in Fig. 10(a), Fig. 10(b), Fig. 11(a), and Fig. 11(b), demonstrate that the FOPID-FFO controller significantly outperforms the conventional PID-PSO controller in mitigating frequency deviations and disturbances. Furthermore, as shown in Table 5, the proposed method achieves an optimized fitness value of 0.44, compared to 0.53 for the traditional PID-PSO technique, confirming its superior stability performance under fluctuating loads.

**Table 5:** Fitness value for case-1.

	Case -a	Case-b
Fitness value	0.44	0.53

**Fig. 12:**
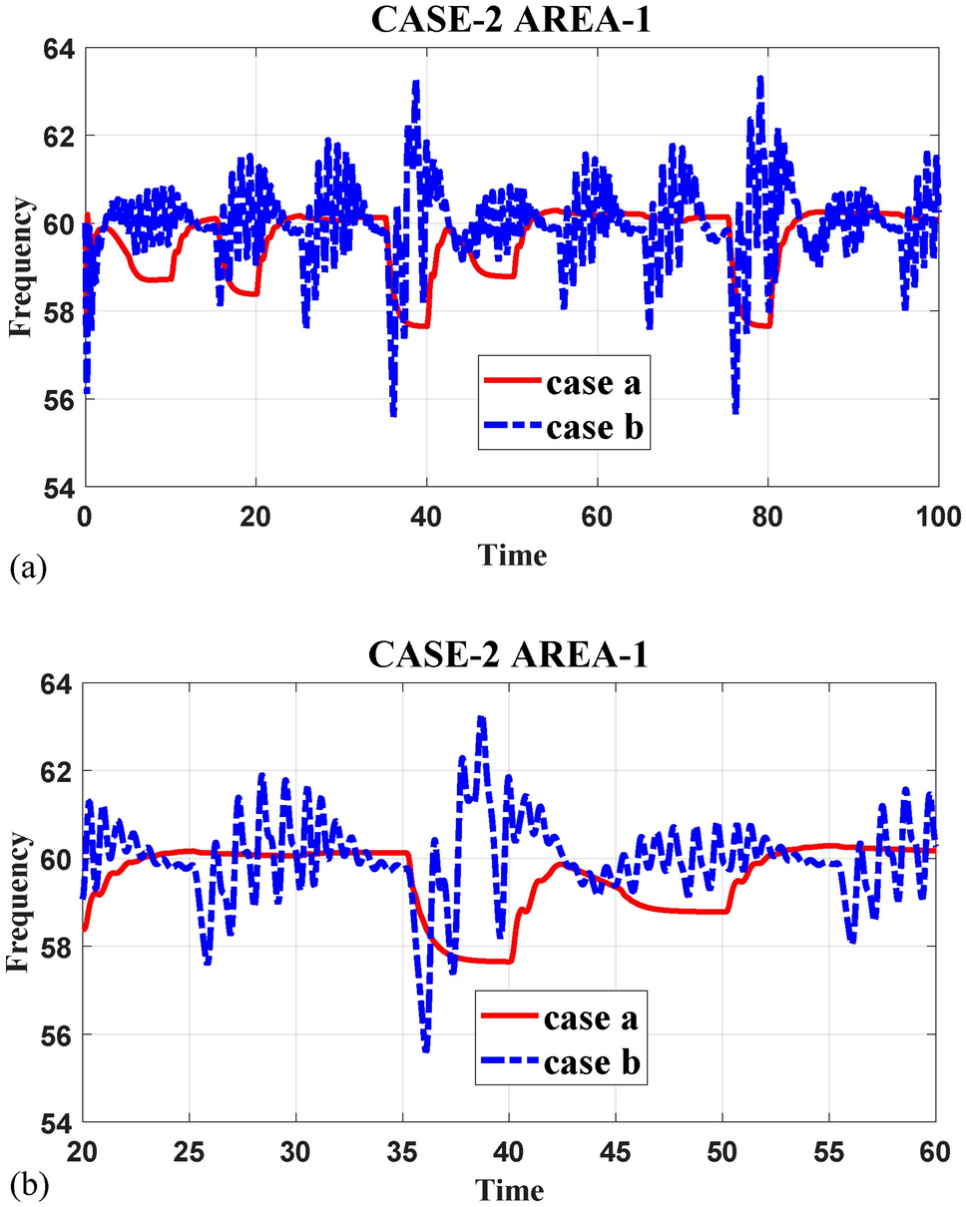
(**a**) Frequency-time response for area-1 under case-2 (**b**) Zoomed in frequency-time response for area-1 under case-2.

**Fig. 13:**
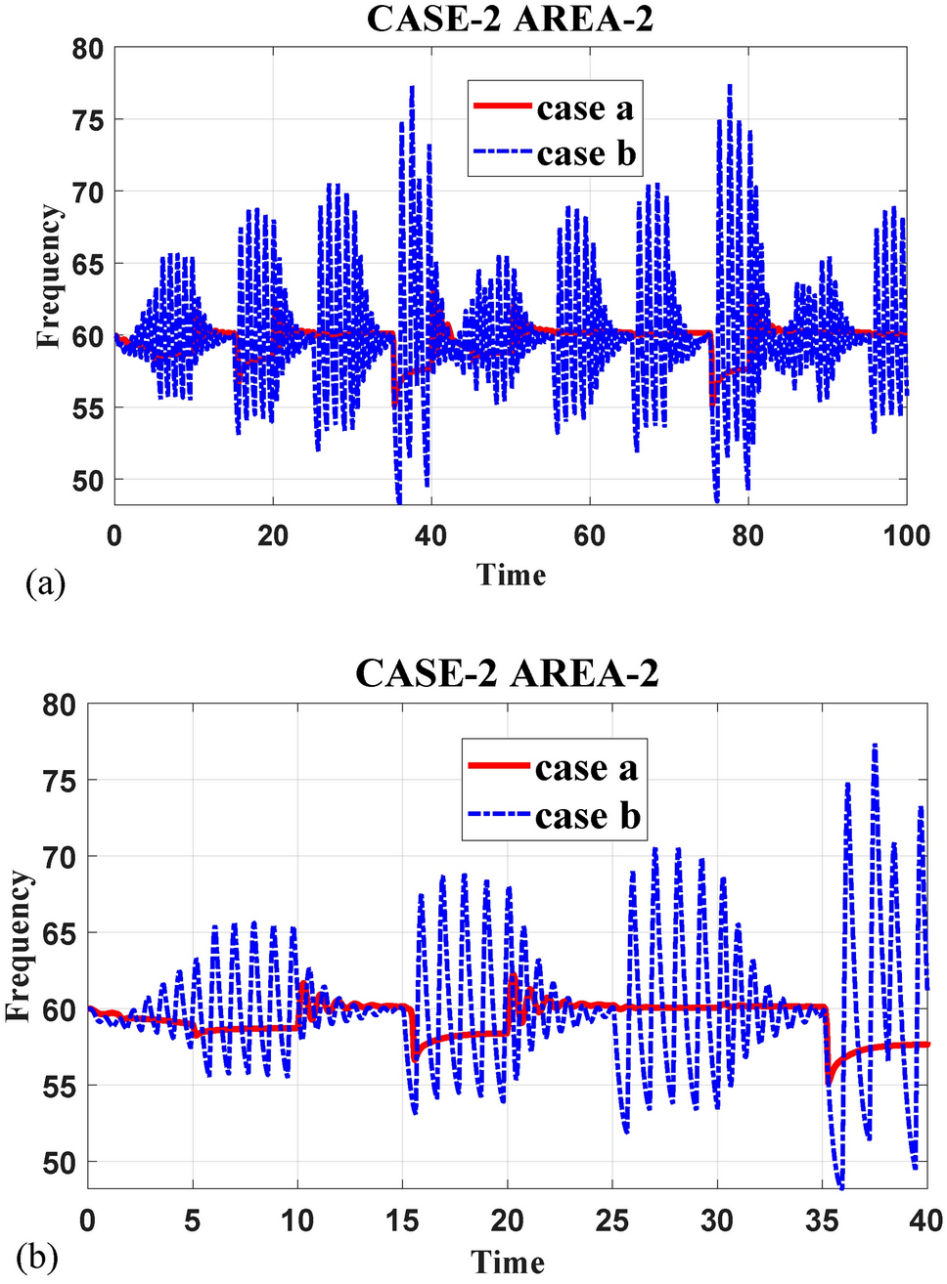
(**a**) Frequency-time response for area-2 under case-2 (**b**) Zoomed in frequency-time response for area-2 under case-2.

Similarly, in Case 2, where the system was subjected to variable weather conditions, the frequency response results in Fig. 12(a), Fig. 12(b), Fig. 13(a), and Fig. 13(b) further validate the robustness of the FOPID-FFO controller. The improved fitness value of 398.74 for Case-a (FOPID-FFO), compared to 1352.75 for Case-b (PID-PSO) as per Table 6, highlights the enhanced ability of the fractional-order controller tuned with the firefly optimization algorithm to mitigate renewable energy-induced fluctuations and maintain frequency stability in hybrid power networks. These real-world case studies reinforce the practical applicability of the proposed control strategy in the Indian and African energy markets. The findings confirm that the FOPID-FFO controller improves frequency regulation, enhances system resilience, and ensures stable power system operation in hybrid energy networks with high renewable penetration. Given the growing demand for decentralized energy solutions in rural India and Africa, this methodology provides a scalable and computationally efficient solution for strengthening grid reliability and optimizing hybrid energy integration.

**Table 6:** Fitness value for case-2.

	Case -a	Case-b
Fitness value	398.74	1352.75

The integration of renewable energy sources into power systems has significantly increased in recent years, making it essential to develop efficient strategies for generation control in interconnected power networks. In this context, several optimization algorithms have been proposed to address the challenges of generation control. One such approach is the Chaotic Chimp Algorithm, proposed in^158^, which specifically focuses on improving the performance of generation control in a three-area interconnected power network. This algorithm leverages chaotic maps to enhance the exploration and exploitation phases, ensuring better convergence towards optimal solutions for power generation control.

However, the authors in^159^ took a different approach by modifying the cost function to achieve an effective solution for generation control using the Stochastic Fractal Search method. The alteration of the cost function in^159^ was crucial in enhancing the solution quality while maintaining efficiency in handling the dynamic nature of power generation systems, which often include renewable energy sources like wind and solar power.

Authors of^160^ addresses the critical challenge of LFC in interconnected power systems, particularly focusing on the application of an Event-Triggered (ET) based multi-rate output feedback discrete sliding mode control (DSMC) method. In a two-area power system, maintaining frequency stability and effective power exchange between areas becomes complex under varying load demands and inherent system non-linearities, such as governor rate constraints (GRC) and generation dead band (GDB). By implementing a multi-rate feedback structure, the proposed controller dynamically adjusts the control signal at varying sampling intervals, responding selectively to changes that significantly affect system stability. This flexibility enables the controller to act efficiently under high-load fluctuations, maintaining frequency and tie-line power within permissible limits.

The multi-rate DSMC approach offers a nuanced advantage in managing control efforts within the interconnected system, especially under load disturbances. Traditional control mechanisms may fail to maintain performance when facing non-linearities like GRC and GDB, which often impose limitations on system response. However, the ET-based multi-rate output feedback control addresses these challenges by adjusting control updates based on specific triggering events, avoiding unnecessary computational loads while maintaining system stability. This strategic event-based control activation allows the system to respond more effectively to disturbances and mitigates unnecessary resource utilization, which is critical for real-time applications in power systems.

A thorough comparative analysis was conducted to evaluate the performance of the proposed controller against conventional methods, such as ET sliding mode and PID controllers, under identical operating conditions. The comparison revealed that the proposed ET-based DSMC controller significantly outperforms traditional control schemes in terms of response times, frequency oscillation minimization, and overall robustness. These findings underscore the proposed method’s ability to provide better settling times, reduced overshoot, and improved overall system performance during transient conditions, even when subjected to high non-linearities. Such improvements in control dynamics can lead to more efficient power exchanges, enhancing the system’s resilience and stability, which is essential in handling real-time power demands and sudden disturbances.

The ET mechanism embedded within the controller also addresses one of the critical challenges in networked control systems: reducing communication load without compromising performance. Frequent data exchange in power systems can cause delays, which may degrade performance. The ET method reduces the frequency of data transmission by activating control updates only when necessary. This not only lowers communication demands but also optimizes network bandwidth usage, which is particularly advantageous in modern, smart grid applications where efficient data management is crucial for maintaining stable operations. Additionally, the method’s ability to handle non-linearities and disturbances while minimizing data transfer aligns well with the needs of advanced power networks, contributing to system-wide robustness.

In summary, the study^160^ demonstrates that the proposed ET-based multi-rate output feedback DTSMC controller is highly effective for LFC in two-area power systems, providing enhanced stability, robustness, and resource optimization. The unique combination of ET and multi-rate feedback allows for efficient real-time control, addressing both the system’s dynamic demands and practical communication limitations. The proposed method is well-suited for expanding applications in future power systems, especially as grids become more complex and data-driven. Moving forward, this controller could be adapted to systems with larger interconnections and time-delay elements, further strengthening frequency stability in next-generation, highly responsive power networks.

Authors of paper^161^ presents an advanced, data-driven cooperative LFC strategy designed for multi-area power systems, addressing both dynamic load conditions and the heightened vulnerabilities posed by cyber-attacks. By integrating Voltage Source Converter (VSC) models and Electric Vehicle (EV) aggregators, this approach establishes a robust framework for dynamic frequency regulation. Through a space-vector-based transfer function, it accurately maps the relationship between active power deviations and angular frequency variations, thereby facilitating effective control responses. The inclusion of VSCs and EV aggregators introduces multiple layers of adaptability, enhancing the resilience and responsiveness of LFC mechanisms under varying operational scenarios, including those with disruptive cyber interference.

A Multi-Unit Deep Reinforcement Learning (MUDRL)^161^ framework is employed to manage the complex, decentralized nature of the system. Agents are strategically positioned across control zones, where they undergo centralized training in a simulated LFC environment designed to maximize a reward function aimed at frequency stability, precision, and robustness. This training equips each agent with an optimal control policy, while a delayed policy update mechanism mitigates the adverse effects of cyber-attacks on control responses. The MUDRL agents collaborate across thermo-turbine-driven generators, VSC-based sources, and EV aggregators, ensuring smooth coordination and effective load balancing in real-time. Such a decentralized, cooperative approach is crucial in modern power networks, where ensuring system stability under diverse and uncertain conditions is increasingly challenging.

Quantitative analyses confirm the superiority of the proposed MUDRL-based control strategy over traditional model-based approaches. This advanced method not only achieves high-precision frequency regulation that competes with strategies based on physical models but also demonstrates robust resilience against cyber-attacks, effectively minimizing frequency deviations and oscillations. In real-time applications, this method offers faster response times, reduced overshoot, and improved control accuracy, reflecting its robustness and practicality in fluctuating operational environments. These findings affirm the method’s suitability for large-scale deployment in complex, interconnected power systems where resilience, adaptability, and precision are paramount.

The cooperative, data-driven control structure of the MUDRL framework provides agents with the capability to make highly adaptive, real-time control decisions that uphold frequency stability amidst uncertainty. By dynamically adjusting control policies, each agent autonomously executes optimal actions that contribute to maintaining system stability, even under cyber-attack conditions. This approach not only enhances the efficiency and security of LFC in modern power systems but also represents a significant step forward in intelligent, data-centric control solutions for power system engineering. The demonstrated improvements in response time, control precision, and system resilience validate the effectiveness of the MUDRL-based strategy, offering a robust foundation for future research in intelligent LFC systems.

To further advance the proposed LFC framework in^161^, several promising areas of research warrant exploration. First, extending the VSC model to incorporate voltage regulation alongside frequency control could create a more unified and comprehensive power system stability framework. Additionally, developing a multi-objective optimization approach that combines insights from both cyber and physical layers would provide a more holistic view of power system control, balancing reliability with economic considerations. Another key area is the integration of cybersecurity protocols directly into the LFC control architecture, particularly through adaptive MADRL-based mechanisms that can identify and counteract cyber-attacks in real-time. Furthermore, exploring hybrid reinforcement learning methods, such as combining MUDRL with transfer learning, could enable faster convergence and improved performance in new, previously unseen operational scenarios. Lastly, research into the integration of this framework with distributed energy resources, such as renewable energy sources and storage systems, would expand its applicability in highly variable, low-inertia power systems. Together, these research directions have the potential to significantly enhance the resilience, efficiency, and adaptability of cooperative control strategies in the future of smart, interconnected power systems.

One of the primary goals in the development of optimization algorithms for generation control in interconnected power networks is the reduction of computational complexity. This is crucial because computational complexity directly impacts the time taken for each iteration of the optimization process, and ultimately influences the overall time required to converge to an optimal solution. In power systems with high integration of renewable energy sources such as solar, wind, and biomass, minimizing computational load becomes particularly important due to the real-time nature of power system control. Faster and more efficient algorithms allow for quicker decision-making, which is vital in systems where power generation is dynamic and unpredictable.

However, while minimizing computational complexity is a common objective, there are inherent challenges in hybrid power networks that can limit how much complexity can realistically be reduced. Hybrid power systems, which combine conventional and renewable energy sources, present a range of non-linearities and uncertainties that are difficult to model and manage. The variability in green energy sources, particularly in solar and wind power, introduces fluctuations in power generation that require sophisticated control mechanisms. Therefore, reducing computational complexity alone is not always sufficient to address the broader challenges of managing hybrid networks.

Given these constraints, researchers are increasingly exploring ways to mitigate the added complexity by leveraging specific properties of renewable energy sources. Instead of solely focusing on simplifying the computational models, new approaches are being considered that use inherent characteristics of renewable energy to balance or counteract the complexity introduced by other energy sources. For instance, the relatively predictable nature of solar energy during clear weather conditions or the long-term seasonal patterns of wind generation can be used strategically to offset the unpredictability of other renewable sources or to simplify certain aspects of the control model.

By identifying these stabilizing properties within green energy sources, it may be possible to create algorithms that are better optimized for hybrid networks. For example, incorporating stochastic models that recognize the recurring patterns in certain types of renewable generation could reduce the need for constant recalculations or adjustments in the control process. Similarly, using energy storage systems, like batteries, to buffer the variability of renewable sources can provide a smoothing effect that reduces the overall complexity of generation control.

Furthermore, these properties could be integrated into the design of optimization algorithms in a way that promotes a balance between complexity and stability. Instead of relying on computational shortcuts, researchers could focus on developing models that use the predictable behavior of renewable energy to compensate for the variability or uncertainty in other parts of the network. This approach may involve a combination of dynamic control strategies, predictive modeling, and energy management techniques, which together can result in a more resilient and efficient hybrid power system.

In conclusion, while reducing computational complexity remains an important objective for improving the real-time control of hybrid power networks, it is not always feasible to achieve significant reductions due to the inherent complexity of renewable energy systems. Researchers must look beyond just computational efficiency and consider the properties of renewable energy itself as a means of counteracting and balancing the complexity introduced by other sources. By leveraging the stabilizing characteristics of green energy, new, more effective approaches to generation control can be developed, leading to enhanced system performance and reliability in hybrid power networks.

With the rapid advancements in innovation and automation across sectors, the power industry, particularly in India and African nations, faces critical challenges in meeting the escalating energy demands, which are compounded by the vulnerability of grid systems to major blackouts. In both India and many African countries, frequent and prolonged blackouts are often caused by issues such as aging infrastructure, inadequate grid capacity, and the intermittent nature of renewable energy sources. Addressing these challenges requires the adoption of advanced energy generation strategies, robust grid management, and the development of resilience frameworks to minimize the risk and impact of blackouts.

This review paper examines these issues, focusing on the integration of renewable energy sources like nuclear and wind with conventional power systems. It delves into how blending these diverse energy sources within existing grid structures can strengthen generation control and enhance the reliability of power supply. Such integration is crucial for both India’s rapidly growing grid and the evolving energy systems in many African countries, where grid reliability and power availability remain significant concerns.

One of the major challenges that both regions face is the increasing frequency of grid blackouts. In India, instances like the 2012 blackout, which affected over 600 million people, underscore the vulnerabilities inherent in the power grid system, especially as demand grows and renewable energy penetration increases. Similarly, many African countries suffer from persistent outages due to underdeveloped infrastructure and a heavy reliance on unstable power generation methods. These blackouts often result in severe economic disruptions, social unrest, and hindered development.

The study investigates the impact of fluctuating load conditions, environmental factors, and aging infrastructure on grid resilience. It emphasizes the need for advanced optimization and control strategies to ensure the stability of power grids and reduce the risks of blackouts. The review explores how optimization frameworks, including metaheuristic algorithms like TLBO and PSO, can improve grid performance, manage fluctuations, and enhance system resilience in the face of disruptions. These techniques can also help predict and mitigate the likelihood of blackouts by optimizing grid operations in real time.

The growing reliance on offshore renewable energy sources such as wind and tidal power necessitates advanced strategies for LFC and AGC to address the operational challenges posed by their inherent variability. Offshore power systems operate in dynamic marine environments where fluctuations in resource availability and harsh weather conditions impact grid stability. Effective control strategies are essential to mitigate these challenges, ensuring robust frequency regulation and reliable power delivery in interconnected grid systems. To achieve this, a combination of advanced controllers, predictive techniques, energy storage solutions, and optimization-based designs has been employed to enhance the technical robustness and adaptability of LFC and AGC frameworks.

Decentralized and hierarchical control architectures have proven effective in offshore power systems by enabling local controllers to independently manage frequency regulation, thereby reducing communication delays and dependency on centralized systems. These architectures are often supplemented with hierarchical frameworks, where lower-level controllers address local frequency deviations and higher-level controllers manage coordination across offshore and onshore grids. This multi-layered approach enhances operational resilience and flexibility. FO controllers have also emerged as a preferred choice for LFC and AGC in offshore environments due to their ability to model and control complex system dynamics with greater precision. These controllers outperform conventional integer-order designs, particularly in handling the nonlinear and uncertain behaviour of offshore renewable energy sources.

Intelligent control methods, including Artificial Neural Networks (ANN), Fuzzy Logic Systems (FLS), and hybrid systems, further enhance the robustness of offshore power systems. These controllers employ adaptive algorithms to optimize power generation and frequency regulation in real-time, ensuring seamless responses to resource and load fluctuations. Predictive control strategies, such as MPC^162^, leverage resource estimation and forecasting techniques to anticipate system behaviour and pre-emptively optimize control actions. Combined with adaptive control mechanisms, these strategies allow offshore systems to dynamically adjust to changing environmental and operational conditions, significantly improving frequency stability.

ES technologies play a pivotal role in enhancing the performance of LFC and AGC in offshore power systems. High-response systems like batteries, flywheels, and supercapacitors provide immediate frequency support during transient events, while long-duration solutions such as pumped hydro storage and hydrogen-based energy storage systems address sustained generation variability. By integrating energy storage with renewable generation, offshore systems can effectively mitigate short-term fluctuations and enhance overall grid stability. These storage systems are increasingly coupled with advanced optimization techniques, such as GWO and PSO, to fine-tune control parameters for maximum efficiency and reliability.

The use of HVDC transmission systems is integral to the integration of offshore power into onshore grids. HVDC systems, equipped with Voltage Source Converters (VSCs), enable precise control of power flows and frequency regulation. VSC technology offers fast response times, bidirectional power transfer, and efficient multi-terminal configurations, making it ideal for large-scale offshore power networks. Furthermore, coordinated multi-area control strategies allow offshore power systems to operate as part of interconnected grids, distributing generation adjustments across regions to maintain frequency stability while optimizing resource utilization.

Future advancements in offshore power systems, such as hybrid platforms combining wind, tidal, and wave energy, and the deployment of grid-forming inverters, will further enhance the reliability of LFC and AGC frameworks. The integration of blockchain technology for decentralized energy trading and advanced cybersecurity measures will also bolster system resilience. By leveraging these innovative solutions, offshore renewable energy systems can overcome their inherent operational challenges and contribute significantly to the global transition toward a sustainable energy future.

The paper also explores the integration of nuclear and wind energy within hybrid power systems, highlighting two core challenges: ensuring economic feasibility and environmental sustainability, while addressing the risk of blackouts. Nuclear energy, while offering high reliability and low emissions, requires stringent control mechanisms to integrate it into complex power systems. These control mechanisms must ensure that nuclear plants operate safely and efficiently, reducing the risk of cascading failures that could lead to large-scale blackouts. Wind energy, on the other hand, is inherently intermittent and subject to fluctuating environmental conditions, which can destabilize power grids if not managed effectively. Advanced control strategies, such as real-time data analytics, machine learning, and soft computing algorithms, are explored as solutions to mitigate these challenges by enhancing predictability, stabilizing grid operations, and preventing blackouts during periods of low wind generation.

To mitigate blackouts, the study proposes several strategies tailored to the specific needs of India and African countries. These include:

Enhanced Grid Infrastructure and Smart Grids: Both regions would benefit from upgrading their aging grid infrastructure and transitioning to smart grids. Smart grids can dynamically adjust to changing demand and generation conditions, better integrating renewable energy sources and minimizing the impact of power fluctuations.

Energy Storage Systems (ESS): Investment in energy storage solutions, such as batteries, can help store excess energy generated during periods of high renewable output and release it during low generation periods. This helps stabilize grid operations and reduce the risk of blackouts caused by renewable intermittency.

Demand Response Programs: In both India and Africa, demand response programs can be developed to incentivize consumers to reduce electricity use during peak demand periods or when grid instability is detected. These programs can help balance supply and demand and prevent grid overloads that often lead to blackouts.

Distributed Generation Systems: By deploying distributed energy resources (DERs), such as rooftop solar panels, small-scale wind turbines, and local biogas plants, India and African nations can reduce reliance on centralized power plants and enhance grid resilience. These localized systems can continue to supply power during grid failures, reducing the impact of large-scale blackouts.

Advanced Control Strategies: Implementing advanced control strategies, such as the use of machine learning and real-time optimization, can allow grids to automatically detect faults, adjust power flows, and isolate affected areas during an outage. These techniques are particularly useful in interconnected multi-area power systems, where disturbances in one region can quickly propagate across borders.

Grid Interconnections: Strengthening grid interconnections between regions and countries can provide greater flexibility in managing power supply and demand, enabling energy import/export during periods of shortages or surpluses. Cross-border power trading, particularly in Africa, can alleviate some of the pressure on national grids and reduce the likelihood of blackouts.

As the world moves toward more sustainable and diversified energy strategies, it is crucial to address the complexities associated with hybrid power systems. Optimization algorithms that can accommodate the unpredictable nature of renewable resources, such as wind and solar, are essential for ensuring grid stability. Power system control strategies must evolve to include real-time adjustments and dynamic responses to fluctuations in demand and supply. Future research should focus on developing scalable, robust solutions that combine advanced optimization techniques with real-time data-driven decision-making, such as multi-agent systems enhanced by deep reinforcement learning (DRL).

Finally, integrating cybersecurity measures within optimization frameworks is critical, especially as cyber-attacks pose an increasing threat to grid security. Ensuring the resilience of power systems in the face of both physical and cyber threats is essential for preventing blackouts. By developing hybrid optimization approaches that combine machine learning with traditional methods, power systems can become more adaptive, responsive, and capable of maintaining operational stability, even as energy landscapes continue to evolve. Such innovations will be key to addressing the challenges of energy access, grid reliability, and blackout prevention in India and African nations, ensuring a more resilient and sustainable future for their power sectors.

## Conclusion

This study investigates advanced optimization techniques and control strategies that are essential for addressing the intricate challenges of modern energy systems. The application of resilient and adaptive control methodologies is particularly critical in the context of interconnected multi-area power systems, which may incorporate a range of generation sources such as thermal, nuclear, wind, and renewable energy technologies. The research focuses on leveraging sophisticated optimization frameworks, like metaheuristic algorithms, to enhance the efficiency, reliability, and sustainability of power networks under dynamic load conditions and external disturbances. As demonstrated through various studies, these optimization techniques, including TLBO and PSO, have proven effective in tuning control parameters for real-time operation of power systems, managing fluctuations, and improving overall system performance. Despite these advances, significant gaps remain in real-world application due to challenges like climatic uncertainties, diverse energy integration, and the need for responsive, real-time adaptability.

A critical examination of the integration of diverse energy sources, particularly nuclear and wind energy, within hybrid power systems, exposes two core challenges: achieving economic viability and ensuring environmental sustainability. Nuclear energy offers high reliability and low emissions but necessitates robust and secure control strategies to manage its integration into complex power systems. These systems require rigorous safety mechanisms and optimized scheduling to prevent failures and ensure stable operation. On the other hand, wind energy presents unique challenges due to its intermittent nature, heavily influenced by environmental factors. To address these issues, advanced control strategies incorporating machine learning, real-time data analytics, and soft computing algorithms have been explored. These methods help optimize energy capture, improve the predictability of generation, and enhance the stability of power grids, even in the face of high variability in wind energy availability.

The review underscores the need for continued innovation in optimization and control strategies to tackle the evolving demands of hybrid power systems. As the world moves toward more sustainable and diverse energy generation methods, the focus must shift to the dynamic integration of these sources into national and global grids. Optimization algorithms that can accommodate the unpredictable nature of renewable resources, such as wind and solar, are crucial to achieving grid stability. Furthermore, power system control strategies must evolve to address the complexities associated with hybrid grids, including the need for real-time adjustments, balancing economic factors, and minimizing environmental impacts. For example, the development of multi-objective optimization frameworks, combining elements of both cyber and physical layers of power systems, holds the potential to improve efficiency and reduce costs while maintaining grid reliability.

To further refine these strategies, future research should focus on scalable and robust solutions that combine advanced optimization techniques with real-time data-driven decision-making. A promising direction is the application of multi-agent systems, enhanced by deep reinforcement learning (DRL), to handle the decentralized nature of modern power grids. These agents can make autonomous, data-driven decisions to adjust control policies and ensure system stability in the face of disturbances. Another critical research area involves enhancing cybersecurity measures within these optimization frameworks, especially as cyber-attacks pose a growing threat to the security and stability of power systems. Furthermore, integrating distributed energy resources (DERs), such as batteries and solar power systems, with advanced optimization algorithms can significantly improve the flexibility and responsiveness of power grids.

Finally, the development of hybrid optimization techniques that combine machine learning with traditional optimization methods may provide faster convergence and more accurate predictions in previously unseen operational conditions. Such hybrid approaches can be particularly useful for systems that are continuously evolving, such as microgrids and smart grids, where operational conditions change rapidly. By developing more sophisticated algorithms that can quickly adapt to new scenarios, researchers can enhance the resilience of power systems to disruptions and maintain operational efficiency even as energy landscapes become more dynamic and complex. This approach, focusing on adaptive optimization and resilient control, will be key to ensuring the long-term sustainability and efficiency of future energy systems.

## Future scope

Looking The evolution of energy systems will be driven by transformative advancements aimed at enhancing efficiency, resilience, sustainability, and frequency stability. Key research directions are poised to shape future power networks, particularly in the Indian and African contexts, where urbanization, energy access challenges, and the shift toward renewables are critical. Advanced hybrid optimization techniques, combining methods like Teaching–Learning-Based Optimization (TLBO) with metaheuristics such as Grey Wolf Optimization and Firefly Algorithms, will address complex, real-time challenges. These approaches will enable scalable and adaptive solutions for diverse load patterns, ensuring reliable operation in regions experiencing rapid demand growth and energy transitions, while addressing the increasing challenge of frequency stability in variable generation systems.

The integration of artificial intelligence (AI) and machine learning will redefine control systems by enabling real-time adaptability to dynamic conditions in multi-area hybrid power networks. These intelligent and resilient control systems will be crucial for managing renewable-nuclear energy mixes, ensuring grid stability amid uncertainties, and addressing frequency fluctuations caused by intermittent renewable generation. Advanced control strategies, such as decentralized frequency regulation and grid-forming inverters, will play a key role in mitigating frequency instability. In regions like India and Africa, such advancements will support rural electrification, microgrid optimization, and enhanced disaster resilience, while providing robust solutions for frequency stability in hybrid and renewable-rich systems.

With the increasing penetration of renewable energy sources such as wind, solar, and hydropower, research will focus on improving their integration through advanced forecasting techniques, adaptive control mechanisms, and energy storage solutions. These solutions will help buffer against variability and ensure grid stability by managing frequency deviations, a crucial aspect of future power systems. Energy storage systems (ESS) and demand response mechanisms will be pivotal in maintaining frequency stability, enabling smooth transitions between generation and load demand. In the context of India and Africa, where energy systems are evolving rapidly, ensuring stable frequency in real-time operations will be crucial for optimizing renewable integration and maintaining system reliability.

Cyber-physical systems (CPS) and Internet of Things (IoT) integration will revolutionize energy monitoring and control, enabling real-time decision-making, predictive maintenance, and anomaly detection, enhancing both grid reliability and frequency stability. These technologies will support the detection of grid disturbances and the automatic activation of corrective measures, such as frequency regulation and load shedding, to stabilize the grid. In India and Africa, cost-effective CPS and IoT deployments will address infrastructure constraints and foster the development of smart grids capable of maintaining stable frequency in complex power networks.

Market-responsive control strategies, including real-time pricing and demand response, will be increasingly important in addressing frequency stability challenges by enabling demand-side management. These strategies will help balance supply and demand, optimize resource allocation, and enhance economic sustainability while ensuring that frequency regulation mechanisms are properly activated during periods of instability. In deregulated markets like India and emerging ones in Africa, such frameworks will support the economic sustainability of power systems while maintaining grid stability.

Environmental sustainability will remain a top priority, with research focusing on minimizing emissions and optimizing the environmental performance of energy systems. Advanced models for emission analysis will guide greener energy solutions, aligning with global climate goals and regional sustainability commitments in India and Africa. Additionally, adaptive pitch angle optimization for wind turbines will enhance energy capture and efficiency, particularly in regions with diverse climatic conditions, reducing the risk of frequency instability caused by low or excessive wind generation.

The seamless integration of multiple energy sources, including renewable, nuclear, and conventional energy, will define the future landscape of power networks. Hybrid systems combining hydrogen, biomass, and advanced storage technologies will ensure efficient and resilient energy systems, providing necessary support for frequency regulation and grid stability. These technologies will be especially critical in mitigating frequency fluctuations in power systems with diverse energy mixes and in regions where grid interconnections are limited.

In India, future research will emphasize grid modernization, microgrid development, and distributed renewable energy integration to meet growing energy demands while ensuring frequency stability in dynamic operating conditions. In Africa, the focus will shift toward decentralized renewables to address energy access challenges, foster rural electrification, and leapfrog traditional energy systems. By addressing regional challenges, leveraging emerging trends, and implementing advanced frequency regulation strategies, Indian and African power networks can achieve greater efficiency, resilience, and sustainability, paving the way for a cleaner, more reliable, and frequency-stable global energy future.

## Data Availability

All data generated or analyzed during this research are contained within the manuscript.
